# Hub Proteins Involved in RAW 264.7 Macrophages Exposed to Direct Current Electric Field

**DOI:** 10.3390/ijms21124505

**Published:** 2020-06-24

**Authors:** Huijuan Li, Shibin Liu, Yongqian Du, Jie Tan, Jiezhang Luo, Yulong Sun

**Affiliations:** 1 School of Electronics and Information, Northwestern Polytechnical University, Xi’an 710072, China; lihuijuan@mail.nwpu.edu.cn (H.L.); duyongqian@nwpu.edu.cn (Y.D.); jietan@mail.nwpu.edu.cn (J.T.); 18829237964@163.com (J.L.); 2 Key Laboratory for Space Biosciences & Biotechnology, School of Life Sciences, Northwestern Polytechnical University, Xi’an 710072, China

**Keywords:** direct current electric field (dcEF), macrophage, RNAs-seq, bioinformatics analysis, molecular dynamics simulation

## Abstract

At present, studies on macrophage proteins mainly focus on biological stimuli, with less attention paid to the responses of macrophage proteins to physical stimuli, such as electric fields. Here, we exploited the electric field-sensitive hub proteins of macrophages. RAW 264.7 macrophages were treated with a direct current electric field (dcEF) (200 mV/mm) for four hours, followed by RNA-Seq analysis. Differentially expressed genes (DEGs) were obtained, followed by Gene Ontology (GO), Kyoto Encyclopedia of Genes and Genomes pathway (KEGG) and protein–protein interaction (PPI) analysis. Eight qPCR-verified DEGs were selected. Subsequently, three-dimensional protein models of DEGs were modeled by Modeller and Rosetta, followed by molecular dynamics simulation for 200 ns with GROMACS. Finally, dcEFs (10, 50, and 500 mV/mm) were used to simulate the molecular dynamics of DEG proteins for 200 ns, followed by trajectory analysis. The dcEF has no obvious effect on RAW 264.7 morphology. A total of 689 DEGs were obtained, and enrichment analysis showed that the steroid biosynthesis pathway was most affected by the dcEF. Moreover, the three-dimensional protein structures of hub proteins were constructed, and trajectory analysis suggested that the dcEF caused an increase in the atomic motion of the protein in a dcEF-intensity-dependent manner. Overall, we provide new clues and a basis for investigating the hub proteins of macrophages in response to electric field stimulation.

## 1. Introduction

As the first defensive line against the invasion of various foreign pathogens of the body, macrophages are strategically distributed in various tissues of the human body, where they phagocytose and process foreign substances, damaged or dead cells, and components in response to various types of stimuli. The integrated feedback of macrophages to various signal stimuli determines the final function of macrophages, which is a very delicate regulatory mechanism. At present, macrophages mainly respond to two major types of stimulation. The first type of stimulation is biological and chemical material stimulation, such as microorganisms, metabolites, and cytokines. Meanwhile, another type of stimulus is physical stimulation, such as electric fields.

Electric fields have effects on a variety of organisms, and promising advances have been achieved, including skin wound repair [1], electrotaxis on cell behavior [2,3], intestinal microbes [4], nematode aging [5], and signaling mechanisms for directional migration of cells [6,7]. Moreover, electric field application-related platforms (microfluidic devices) have also been rapidly developed [8]. Recently, the regulation of macrophages by electric fields, previously uncharted, has become an emerging field [9]. By applying a direct current electric field (dcEF:5–300 mV/mm, 2 h) to human monocyte-derived macrophages, researchers found that macrophages migrate to the anode in an electric field strength-dependent manner, and the electric field significantly enhanced the phagocytic ability of macrophages [10]. Moreover, mouse bone marrow-derived macrophages apparently move toward the anode after being stimulated by a dcEF (300 mV/mm, 4 h) [11]. Very recently, Yaohui Sun et al. showed that a *Salmonella* infection generates an electric field in the gut epithelium of the mouse cecum, which drives the bidirectional migration of macrophages. By using primary mouse peritoneal macrophages (PMs) and bone marrow-derived macrophages (BMDMs), they mimic the infection-generated electric field by exposing macrophages with electric field (dcEF: 400 mV/mm, 3 h), and they also found that *Salmonella* infection switches macrophage galvanotaxis from the anode to the cathode [12]. Taken together, the above studies suggest that the electric field is a new regulatory element that determines the function of macrophages. 

Despite the above studies on the regulation of macrophages by electric fields, the identification of sensitive molecules of macrophages in response to electric fields is considered to be a key breakthrough in understanding the electric field-caused modulation of macrophages. Using such electric field-sensitive molecules as probes, researchers can effectively explore the cellular signaling pathways of macrophages that are exposed to the direct electric field.

Given the importance of modulating macrophage function in situations where electric fields occur, we sought to explore the hub proteins of macrophages exposed to the direct electric field, in particular, the electric field-sensitive molecules and their cellular signaling. The purpose of this study was to: (1) acquire dcEF-sensitive genes by the RNA-Seq method and explore the cell-level signaling pathways of these genes; (2) obtain three-dimensional protein structure models of these sensitive genes; and (3) get structural characteristics of these DEG (differentially expressed gene) proteins on a microscopic scale, such as structural changes. By screening for electric field-sensitive genes and exploring their corresponding signaling pathways, our work has introduced new candidate molecules for macrophage functional regulation, which may provide a new perspective for understanding macrophage signaling regulatory elements.

## 2. Results

### 2.1. Cell Morphology

**Figure 1:**
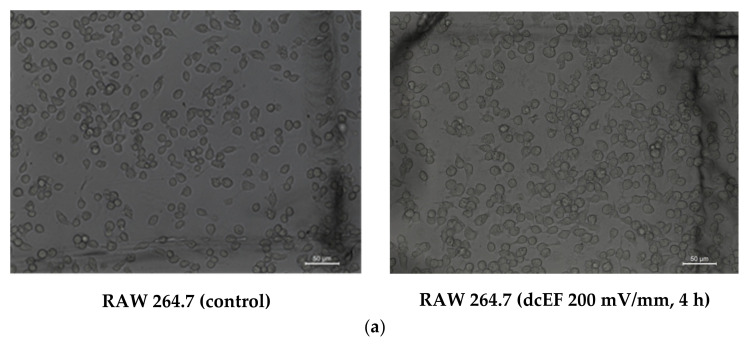

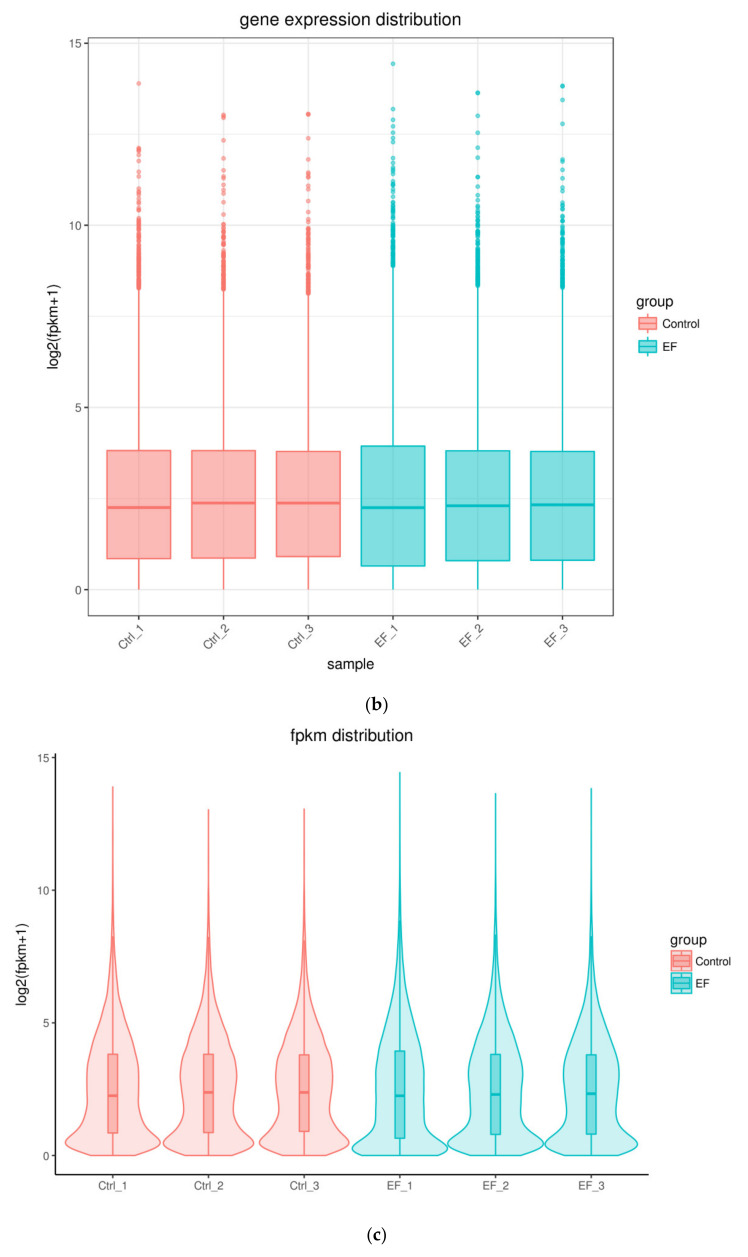

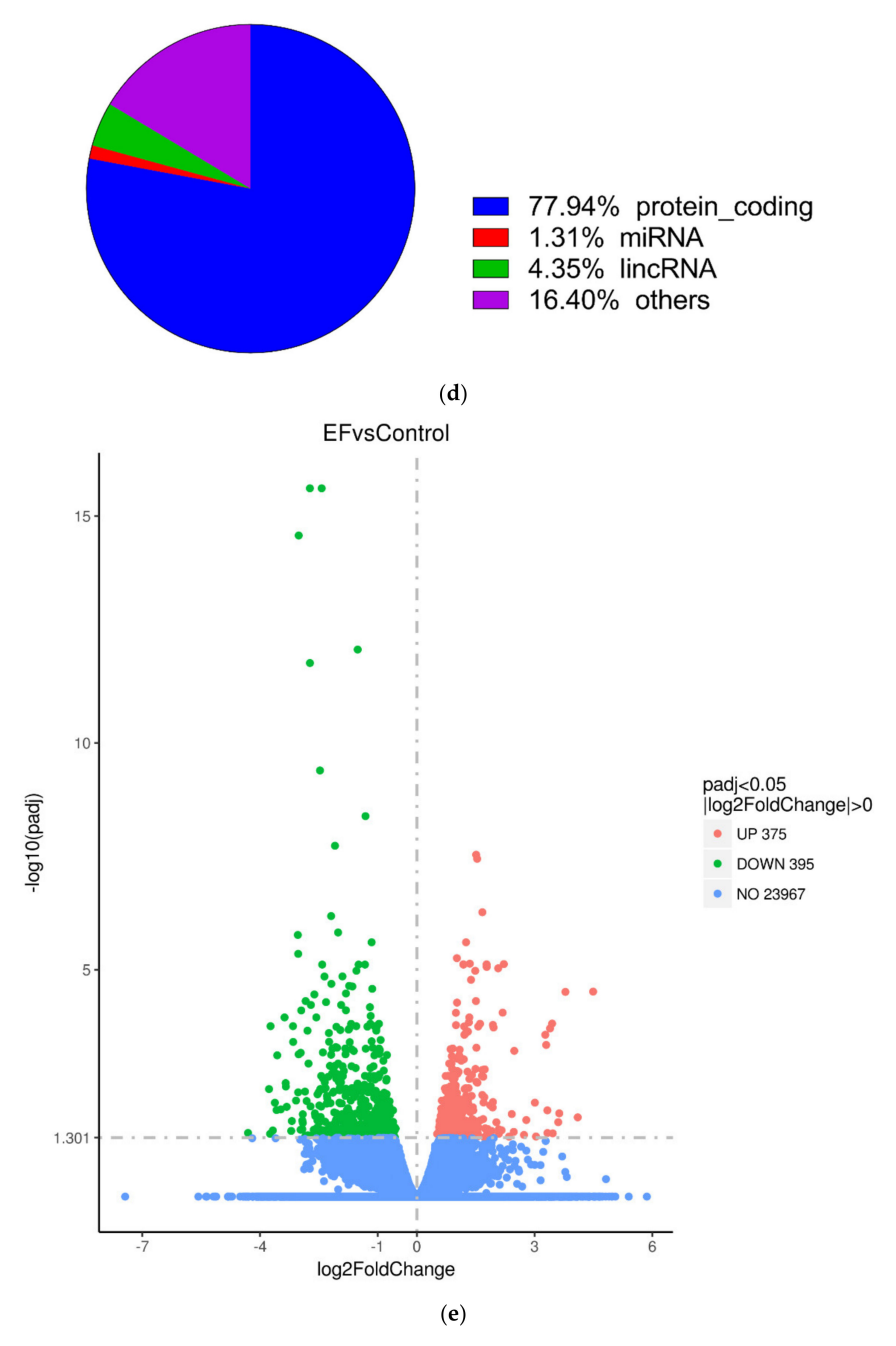

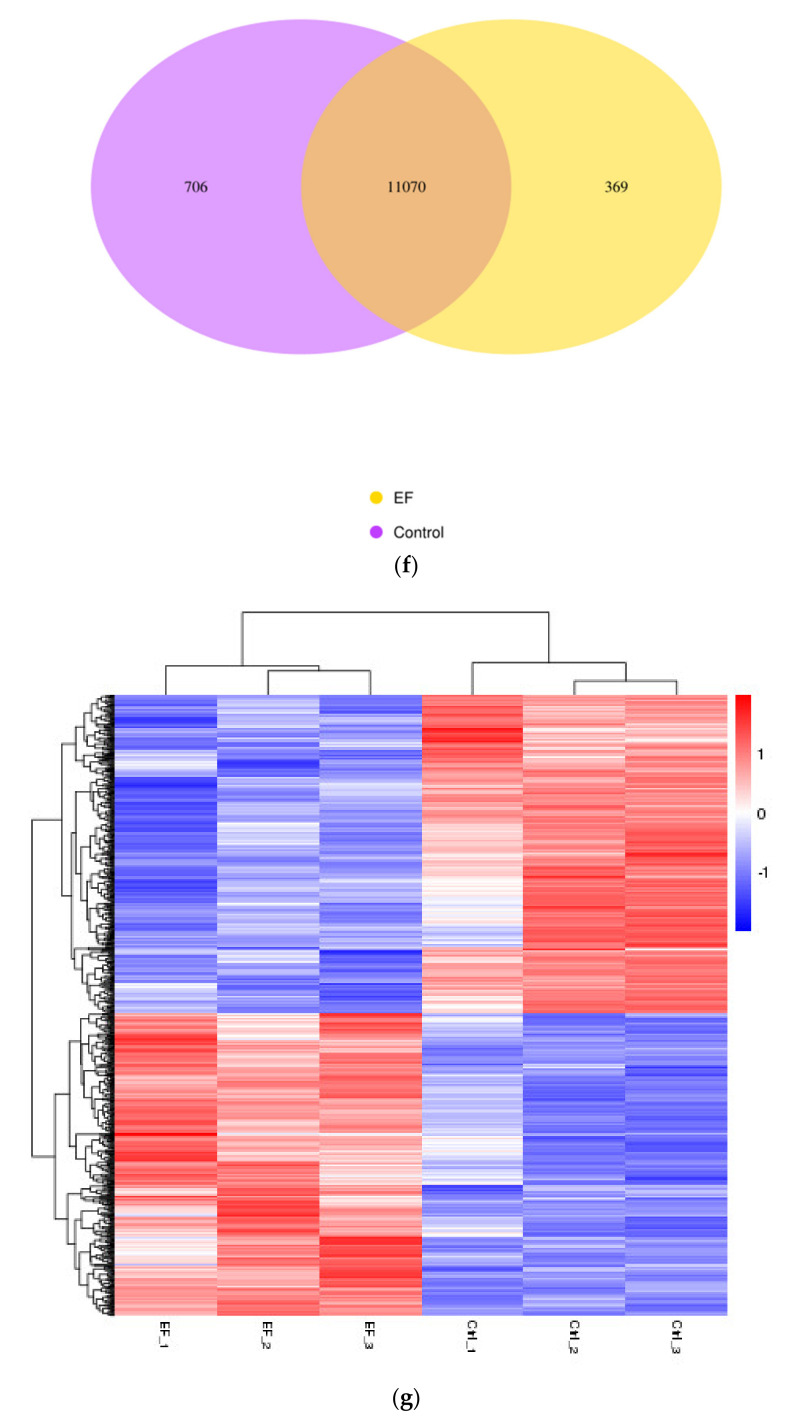
Basic information on RAW 264.7 cells and RNA-Seq. (**a**) The cell morphology of RAW 264.7 cells during the direct current electric field (dcEF) 200 mV/mm treatment. Scale bar, 100 μm. (**b**) The boxplot of gene expression distribution. (**c**) Violin plot. (**d**) The proportion of the sequenced genes. (**e**) Volcano plot showing metabolomic data (up-regulated genes were represented as red dots and down-regulated genes were indicated as green dots). (**f**) Venn diagram showed the number of overlap genes during the different groups. (**g**) Heat map of the differentially expressed genes.

In order to study the effect of dcEF stimulation on cell morphology, the morphological characteristics of cells before and after dcEF stimulation were observed using an optical microscope. Prior to dcEF stimulation, RAW 264.7 cells were mostly oval in shape with clear boundaries and full forms. After treatment at 200 mV/mm for four hours, the cell boundaries were clear and the morphology comprised full forms, suggesting that the dcEF (200 mV/mm) did not significantly cause morphological changes in the RAW 264.7 cells (Figure 1a).

### 2.2. Identification of DEGs 

RNA-Seq was successfully carried out (Figure 1b,c). After RNA-Seq, a total of 689 DEGs were obtained, of which 77.94% were protein-coding genes, 4.35% were Long non-coding RNA (lncRNA), 1.31% were MicroRNA (miRNA), and 16.4% were others (processed_transcript, misc_RNA, and so on) (Figure 1d). The lncRNA and miRNA were screened out from all the samples, and their details are shown in Table 1. The volcano plot in Figure 1e shows the distribution of different genes in each group. The abscissa represents the changes expressed in multiples (log2foldchange) of the gene in the treatment and control groups, and the ordinate represents the high statistical significance between the treatment and control groups (-log10padj or-log10pvalue). The Venn diagram shows that there were 11,070 genes overlapping between the control and EF-treatment groups (Figure 1f,g).

### 2.3. Functional and Pathway Enrichment Analysis of Identified Modules Associated with DEGs

**Figure 2:**
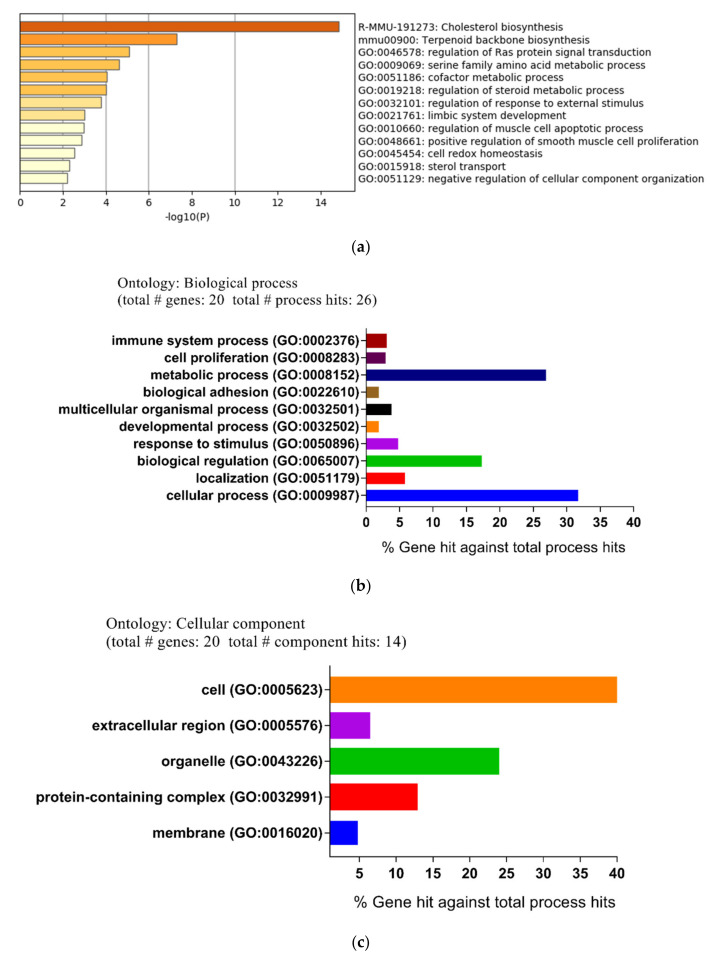

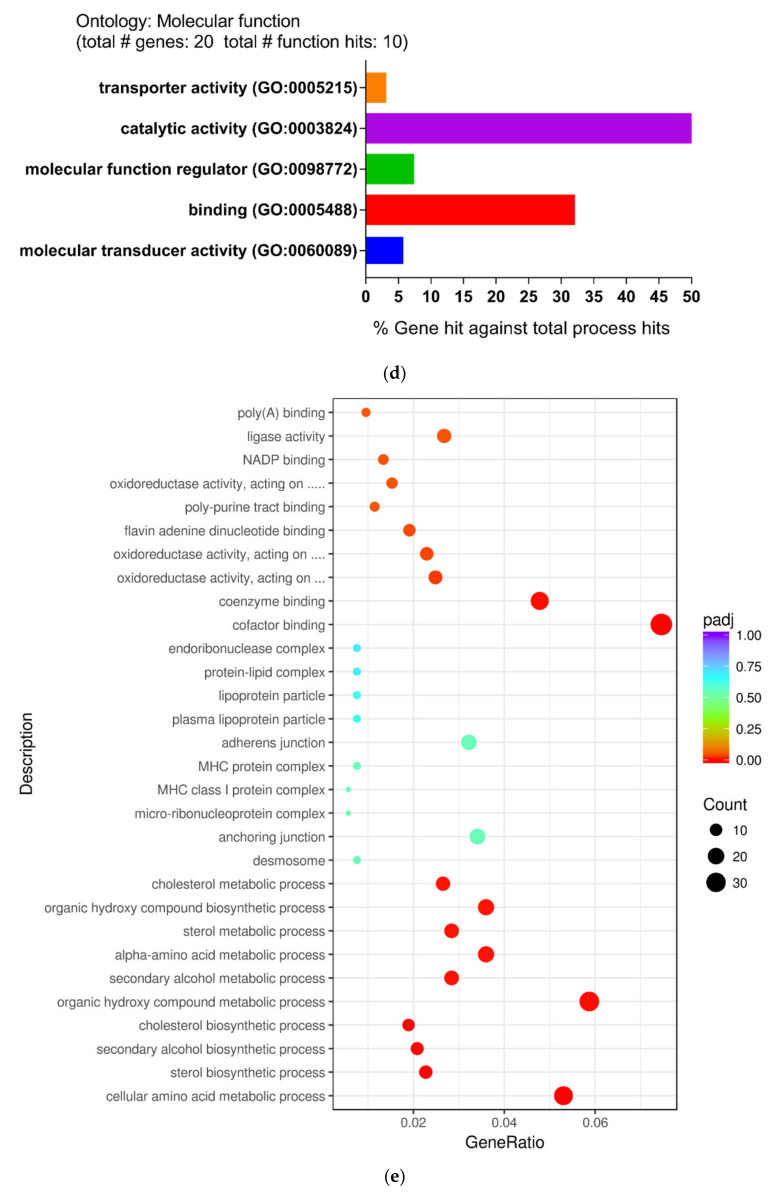
Functional and pathway enrichment analysis of identified modules associated with differentially expressed genes (DEGs). The DEGs were subjected to Gene Ontology (GO) classification using the PANTHER (Protein ANalysis THrough Evolutionary Relationships) GO classification system. (**a**) Bar graph of enriched terms of the genes (colored by *p*-values). (**b**) Biological process (BP). (**c**) Cellular component (CC). (**d**) Molecular function (MF). (**e**) Network maps of GO terms (GO dot). The abscissa is the ratio of the number of differentially expressed genes, which annotates the GO term to the total number of differentially expressed genes.

To identify the function and pathway of these DEGs, an enrichment analysis was performed with the Metascape online tool. All DEGs were uploaded to the online software Metascape to get the pathway and process enrichment analysis. As shown in Figure 2a, the significantly enriched pathways included cholesterol biosynthesis, terpenoid backbone biosynthesis, and the regulation of Ras protein signal transduction. In addition, the enriched terms were connected and clustered into different network plots (Supplementary File 1: Figure S1).

In order to further explore the molecular mechanism and pathways of DEGs, the file of the DEGs was uploaded to the PANTHER (Protein ANalysis THrough Evolutionary Relationships) classification system. Subsequently, the biological process (BP), cellular compartment (CC), and molecular function (MF) were described using the PANTHER classification system. For the biological process, the main differential expression proteins consisted of those of cellular processes (31.7%), metabolic processes (26.9%), and biological regulation (17.3%) (Figure 2b). For the cellular compartment, DEGs were cell (52.4%), organelle (23.8%), and protein-containing complexes (12.7%) (Figure 2c). For the molecular function, the majority of proteins were involved in catalytic activity (52.2%), binding (31.9%), and molecular function regulator (7.2%) (Figure 2d).

Subsequently, the top five Gene Ontology (GO) terms, which were considered the most significant results of GO enrichment, were plotted by network graph. The network diagrams of biological processes, molecular functions, and cellular components were drawn and are shown in Figure 2e. In addition, the five most-significantly enriched pathways of up-regulated and down-regulated DEGs were performed by Kyoto Encyclopedia of Genes and Genomes pathway (KEGG) analysis (Table 2). 

Considering that the steroid biosynthesis pathway was the largest pathway obtained by ClueGO enrichment analysis, this pathway was chosen for subsequent Cytoscape analysis (Supplementary File 1: Figure S2).

### 2.4. Module Screening from the PPI Network

Protein–protein interactions (PPIs) were predicted by using the online STRING database (a database of known and predicted protein-protein interactions). The pictures were constructed and visualized with Cytoscape, followed by module analysis. 

In this study, a total of 48 nodes and 110 edges were identified using the MCODE plugin. The PPI network consisted of three sub-networks, and the module-related genes were then functionally annotated (Supplementary File 1: Figure S3). Enrichment analysis indicated that the genes in modules 1–3 were primarily involved in the cholesterol metabolic process, response to oxidative stress, and the mitotic cell cycle.

**Figure 3:**
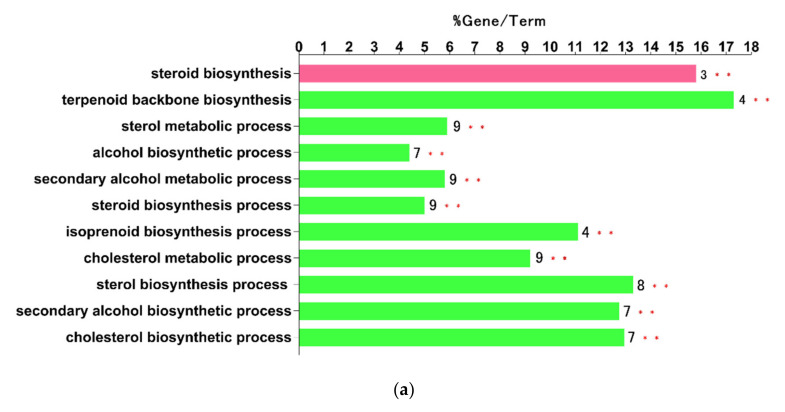

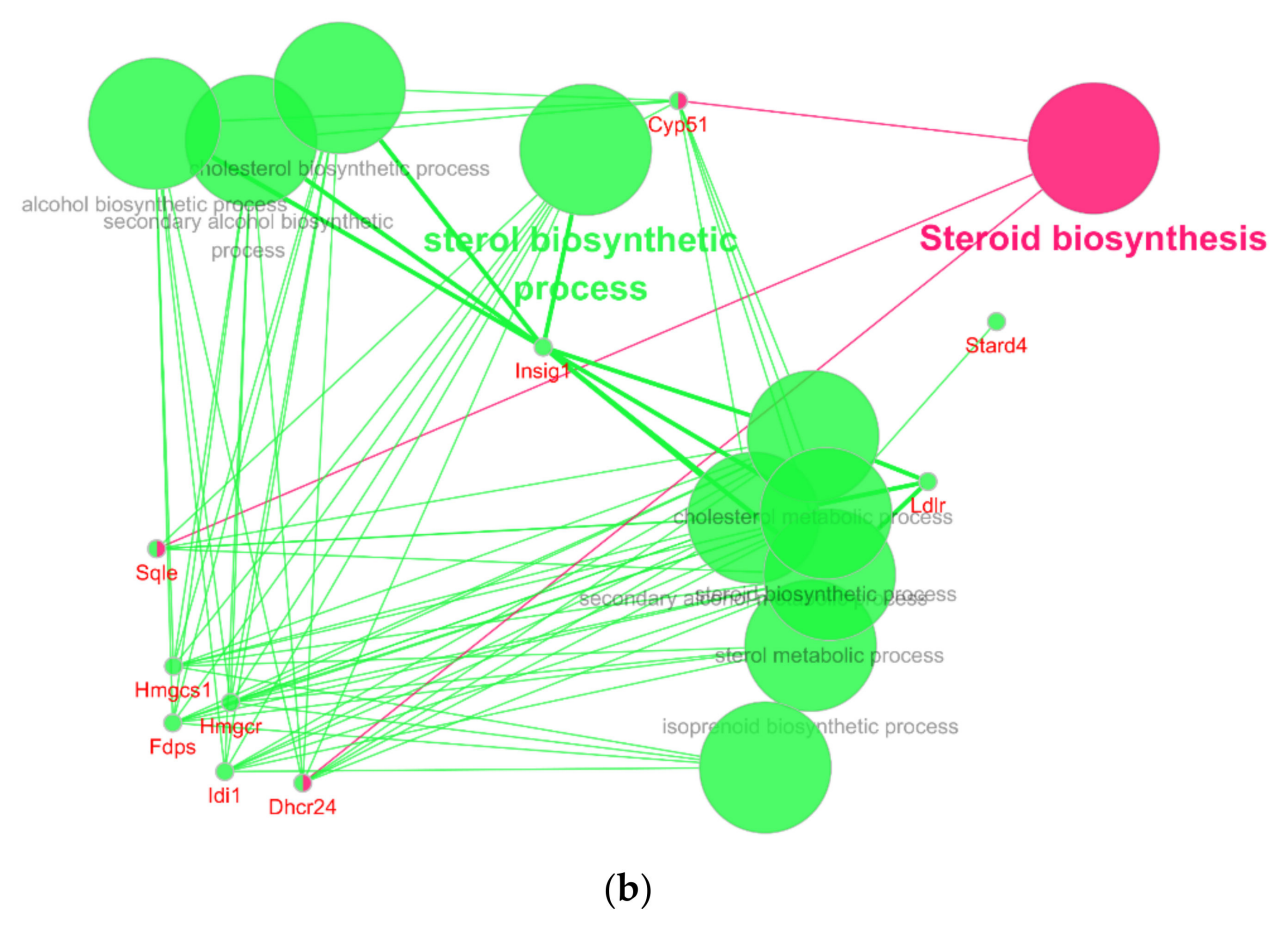
The 10 hub genes were mapped for functional enrichment analysis using the ClueGo tool. (**a**) The enriched pathways of 10 genes (different pathways were represented by different colors). (**b**) Pathway enrichment analysis with ClueGO for the 10 hub genes.

With the help of the cyto-Hubba plug-in of Cytoscape, a total of 10 hub nodes (Cyp51, Fdps, Hmgcr, Hmgcs1, Idi1, Sqle, Insig1, Dhcr24, Ldlr, and Stard4) with higher degrees were obtained. Subsequently, the 10 hub genes were analyzed by ClueGo and CluePedia for functional enrichment analysis. Only pathways with a *p*-value of ≤ 0.05 and a kappa coefficient of 0.4 were considered statistically significant. Eleven pathways could be classified into two categories, as shown in Figure 3a: the sterol biosynthetic process and the steroid biosynthesis (Figure 3b).

### 2.5. Gene Expression Verification of DEGs

**Figure 4:**
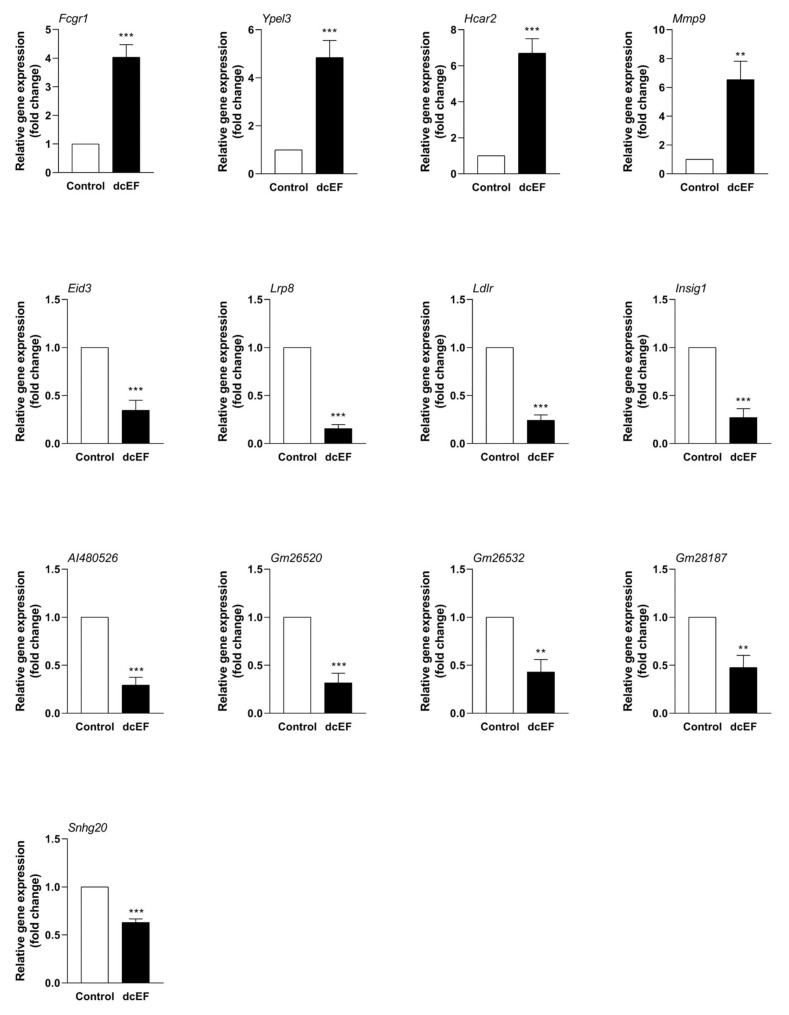
RNA-Seq results were verified by qPCR for DEGs of RAW 264.7 cells in response to the dcEF. RAW 264.7 cells were treated with a dcEF of 200 mV/mm for four hours, followed by gene expression examination. The total RNA was isolated, and a qPCR test was carried out. The mRNA expression was normalized to the expression of GAPDH (Glyceraldehyde-3-Phosphate Dehydrogenase). * significantly different from control; * *p* < 0.05; ** *p* < 0.01; and *** *p* < 0.001. *n* = 3 for each test in duplicate. The final data are shown as means ± SEM, and the statistical significance of results was analyzed by a t-test.

In order to verify the accuracy of the RNA-Seq data, qPCR was performed to further evaluate the effect of the dcEF on the gene expression of the RAW 264.7 cells at the transcriptional level. The genes to be detected mainly include two types: genes encoding protein (Fcgr1, Hcar2, Mmp9, Lrp8, Ldlr, Eid3, Insig1m and Ypel3) and genes encoding lncRNA (AI480526, Gm26520, Gm26532, Gm28187, and Snhg20). As shown in Figure 4, compared with the control group, the trend of all genes was consistent with the RNA-Seq data, where four genes were up-regulated (Lrp8, Ldlr, Eid3, and Insig1) and four genes were down-regulated (Fcgr1, Hcar2, Mmp9, and Ypel3). It is worth noting that all genes encoding lncRNA were down-regulated (AI480526, Gm26520, Gm26532, Gm28187, and Snhg20).

### 2.6. Protein Modeling 

**Figure 5:**
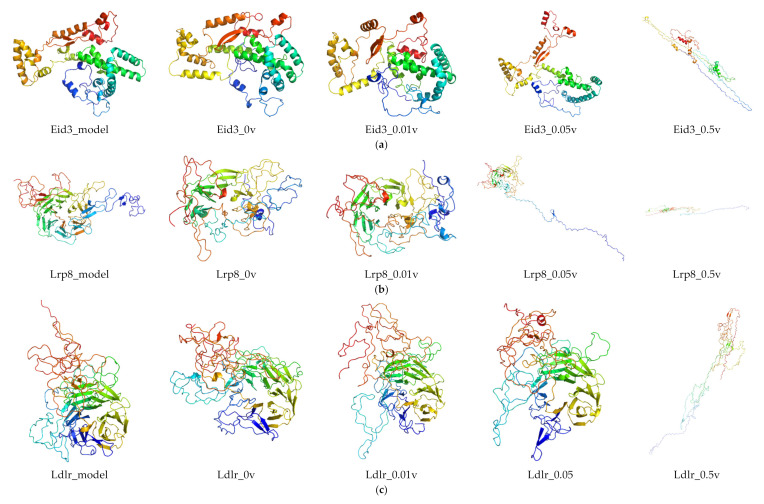

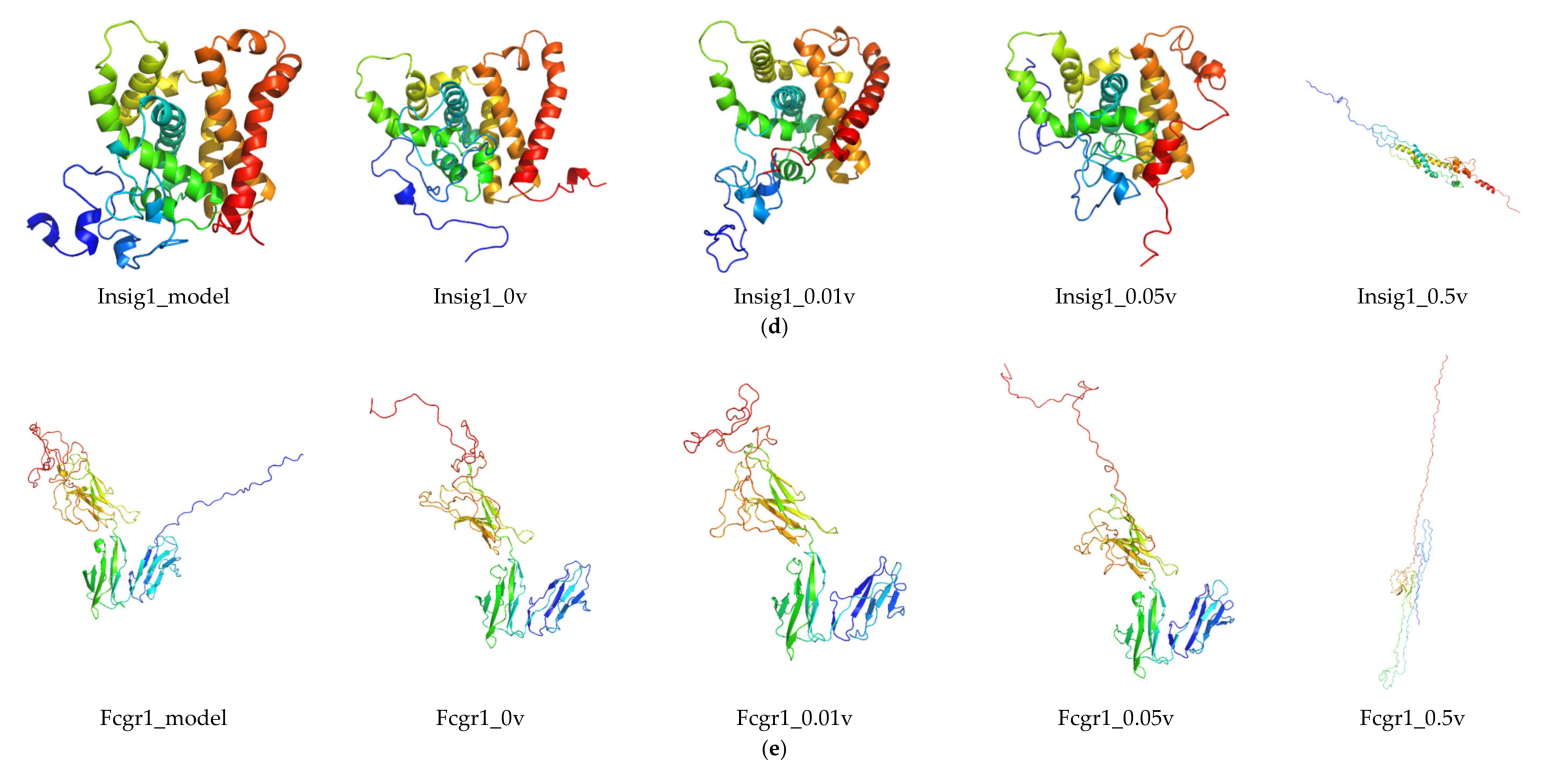

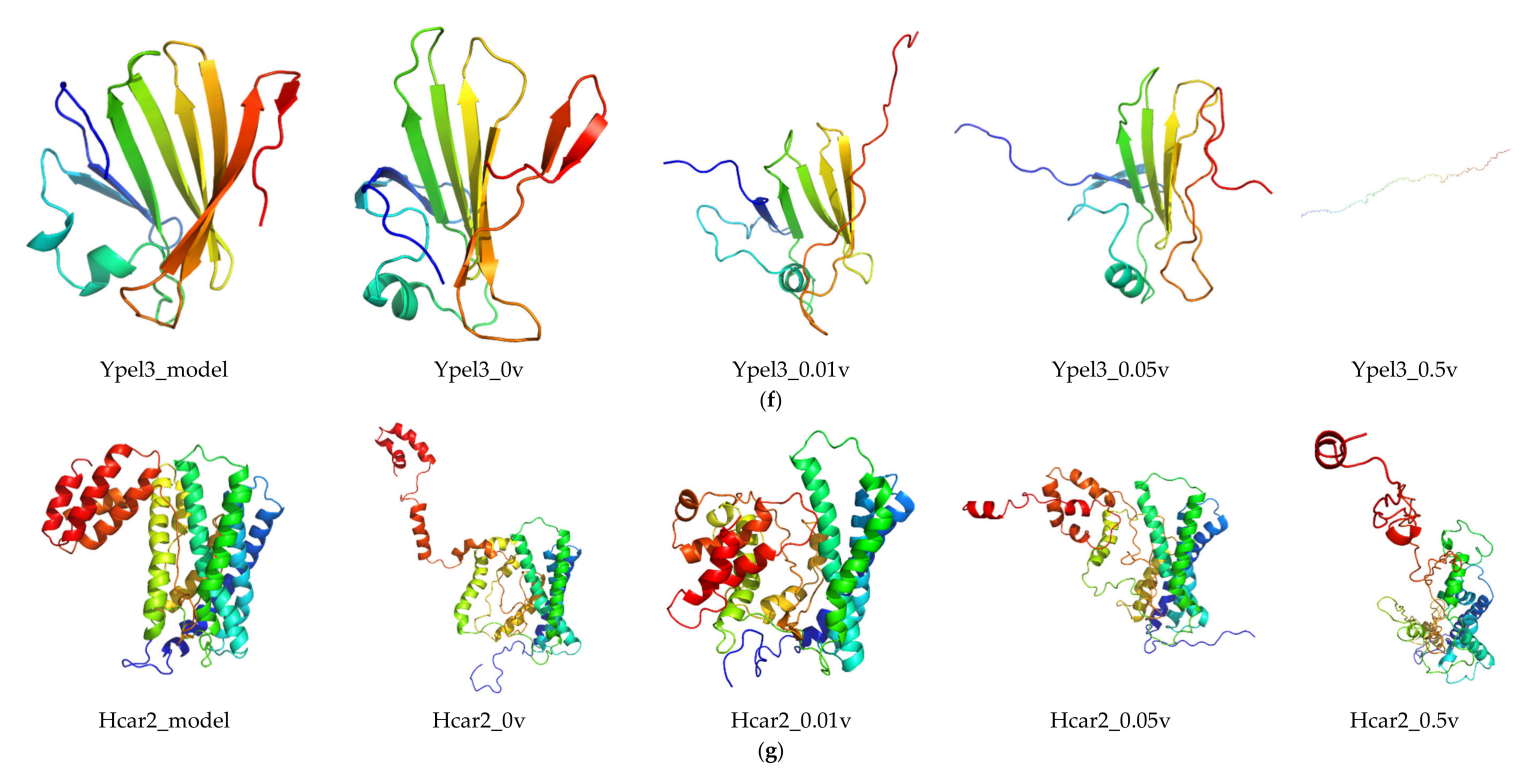

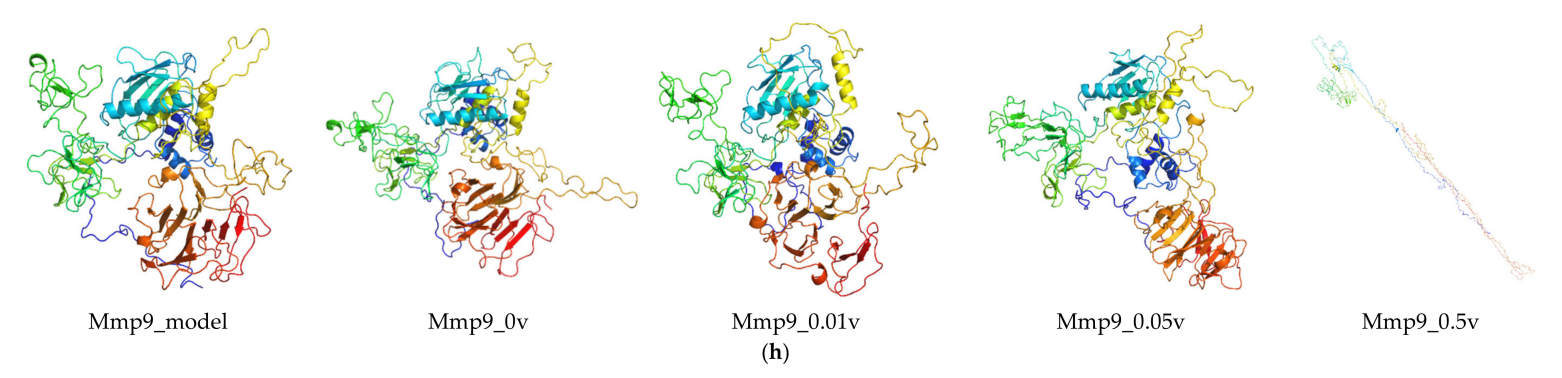
The structure of the three-dimensional protein of DEGs optimized by molecular dynamics. For each protein, from left to right, respectively: three-dimensional protein modeling structure, three-dimensional protein structure of the lowest energy for DEGs after molecular dynamics simulation of 0, 0.01, 0.05, and 0.5 v direct current electric field. Here, the three-dimensional protein structures of Eid3 (**a**), Lrp8 (**b**), Ldlr (**c**), Insig1 (**d**), Fcgr1 (**e**), Ypel3 (**f**), Hcar2 (**g**), and Mmp9 (**h**) are shown. The picture was drawn by the Pymol software (Delano, W.L. The Pymol Molecular Graphics System (2002) DeLano Scientific, SanCarlos, CA, USA. http://www.pymol.org).

In order to further study the protein structure of these DEGs, three-dimensional protein structures of DEGs were modeled by using Modeller (9v21) with multi-template-based protein modeling methods (Figure 5). In all DEG proteins, the three-dimensional protein models of five DEGs were constructed via homology modeling (except for Eid3, Insig1, and Ypel3) (Table 3). The best model for each protein with the lowest discrete optimized protein energy (DOPE) score (Lrp8: −38745.59375; Ldlr: −58098.10156; Fcgr1: −32057.03711; Hcar2: −42692.19531; and Mmp9: −71738.61719) was obtained for further investigation (Supplementary File 1: Figure S4).

Considering the unsatisfactory results from NCBI BLAST analysis, the three-dimensional models of three DEGs (*Eid3, Insig1,* and *Ypel3*) were modeled de novo with the Rosetta Macromolecular Modeling software package. For each protein, 1000 candidate models were generated, and the lowest scored model (*Eid3*: −3.753; *Insig1*: 6.670; and *Ypel3*: −130.395) was selected as the best one for subsequent molecular dynamic (MD) simulation study (Supplementary File 1: Figure S4). 

**Figure 6:**
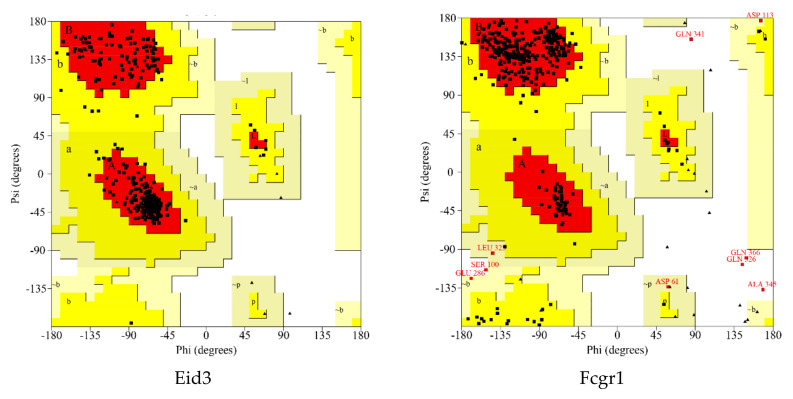

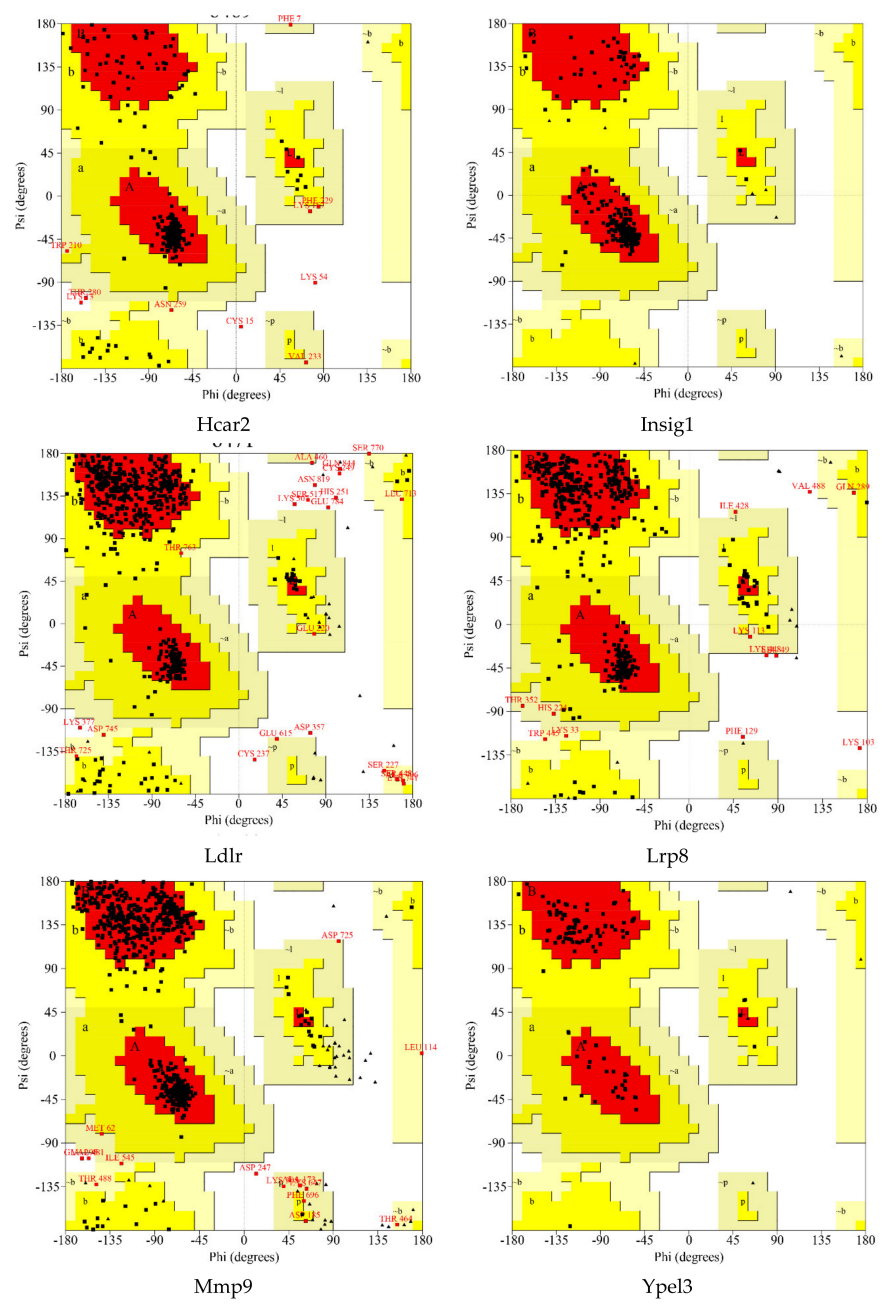
Ramachandran plot of the selected homology modeled 3D protein structures of DEGs. The different colored areas show ‘disallowed’ (beige), ‘generously allowed’ (yellow) and ‘most favored’ (red) regions.

A Ramachandran plot analysis (Figure 6 and Table 4) was conducted to assess the quality of protein modeling. As shown in Figure 6 and Table 4, these residues existed in an outlier region ranged from 0% to 1.7% (LRP8 1.1%, LDLR 1.7%, FCGR1 1.4%, HCAR2 1.2%, MMP9 0.5%, EID3 0.0%, INSIG1 0.0%, and YPEL3 0.0%), indicating that the modeling quality of the protein was acceptable.

### 2.7. Molecular Dynamics and Simulation

**Figure 7:**
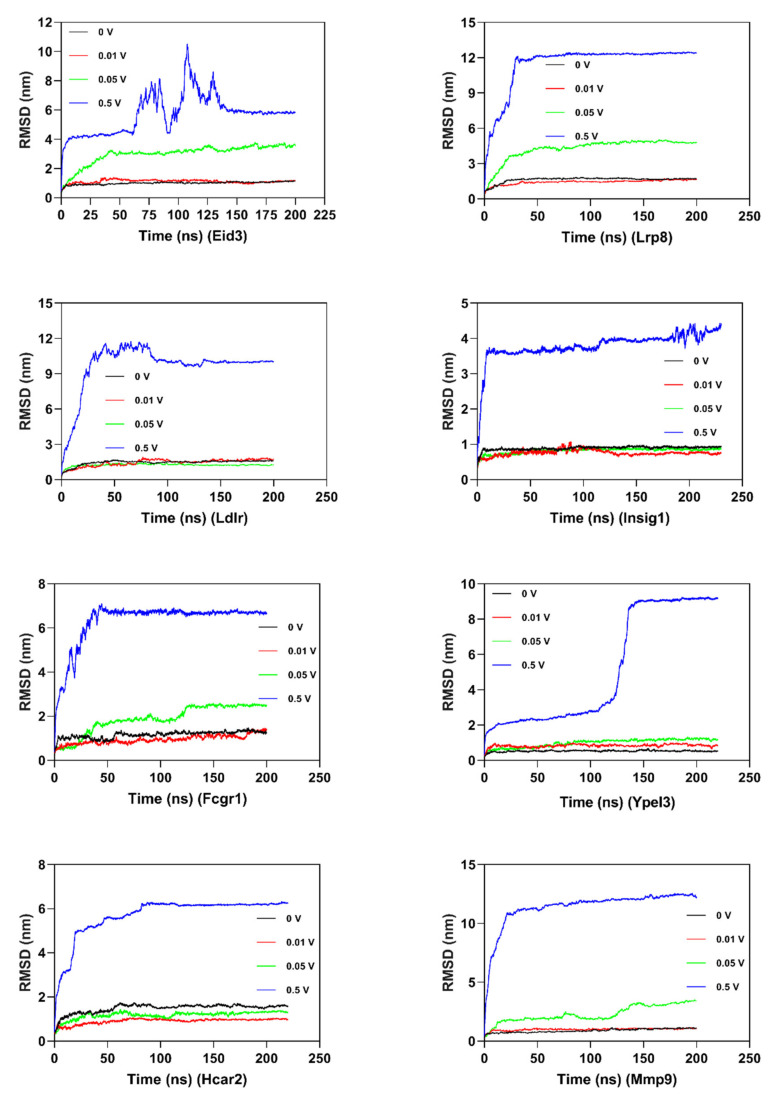
Root mean square deviation (RMSD) comparison plots of backbone Cα atoms during molecular dynamics simulation (at least >200 ns). In order to clearly illustrate the deviations of the DEG proteins, the RMSD plots are shown. The RMSD of *Eid3, Lrp8, Ldlr, Insig1, Fcgr1, Ypel3, Hcar2,* and *Mmp9* are shown. For each protein, 0 mV (black), (red), 50 mV (green), and 500 mV (blue) are displayed on the map.

In order to investigate the structural behavior of DEG proteins on a microscopic scale, MD simulation (at least 200 ns) was conducted for each protein (Figure 7 and Supplementary File 1: Figures S5 and S6). The structural convergence included root mean square deviation (RMSD) (Figure 7), root mean square fluctuation (RMSF) (Supplementary File 1: Figure S5), and gyrate (Supplementary File 1: Figure S6).

RMSD was calculated to analyze the structures and dynamics of the proteins. As shown in Figure 7 and Supplementary File 2: Table S1, the analysis of backbone atoms showed an initial equilibration up to 10 ns (EID3 4 ns, LRP8 20 ns, LDLR 22 ns, INSIG1 4.5 ns, FCGR1 3.4 ns, YPEL3 4.5 ns, HCAR2 14 ns, and MMP9 7.2 ns), and the structures started to converge after different times (EID3 5 ns, LRP8 21 ns, LDLR 23 ns, INSIG1 4.6 ns, FCGR1 3.5 ns, YPEL3 4.6 ns, HCAR2 15 ns, and MMP9 7.3 ns). Subsequently, the models illustrated stable conformation with an RMSD till the end the MD production run (EID3: 0.90–1.01; LRP8: 1.48–1.74; LDLR: 1.38–1.53; INSIG1: 0.82–0.91; FCGR1: 1.00–1.15; YPEL3: 0.42–0.57; HCAR2: 1.22–1.65; and MMP9: 0.69–1.04). When an electric field was applied, the conformational change of the protein was more intense, and this change increased with the increase of the current intensity. Hence, these results indicated that the electric field could significantly affect the atomic motion of the protein in a current intensity-dependent manner.

The RMSF of the atoms of each residue was calculated to explore the flexibility of the protein structure (Supplementary File 1: Figure S5 and Supplementary File 2: Table S2). Low RMSF values of residues suggested less flexibility, whereas high RMSF values depicted more movements during simulation in relation to their average position. As demonstrated in Supplementary File 1: Figure S5 and Supplementary File 2: Table S2, the mean RMSF value of the proteins was between 0.25 and 0.63 (LRP8 0.60, LDLR 0.48, FCGR1 0.45, HCAR2 0.63, MMP9 0.41, EID3 0.38, INSIG1 0.31, and YPEL3 0.25). Similar to RMSD, current stimulation changed the fluctuation of the protein residues, and the amplitude of atomic fluctuation increased with the increase of current intensity. Hence, these results indicated that the electric field-induced protein atom fluctuation enhanced with the increase of electric field intensity.

The radius of gyration (R*g*) demonstrated the level of compactness of the protein structure. The reduction of R*g* values hinted the stability of the system. As shown in Supplementary File 1: Figure S6 and Supplementary File 2: Table S3, the average R*g* value for DEG proteins of 0 mV was from 1.536 to 3.263 (Lrp8 0.16, Ldlr 0.24, Fcgr1 4.03, Hcar2 6.69, Mmp9 6.53, Eid3 0.35, Insig1 1.923, and Ypel3 1.536). The R*g* trend of the 10 mV treatment group was basically consistent with the 0 mV group. As the current intensity increased, the gyration radius of the protein gradually increased (Eid3, Lrp8, Fcgr1, and Mmp9), whereas the R*g* of the other four proteins was basically consistent with the 10 mV treatment group. It is worth noting that in the higher electric field treatment group (500 mV/mm), the protein’s R*g* increased significantly to 4.5–14, hinting a significant decrease in the stability of the simulated system. 

After the MD production run, three-dimensional models of MD-optimized protein models were created (RMSD of primary models and MD-refined models in Figure 5 and Supplementary File 2: Table S4). For most proteins, an increase in current caused a significant increase in the structural changes of the protein. Overall, these results implied that the stability of the protein gradually decreased as the current increased, indicating that current stimulation increased the movement of protein.

## 3. Discussion

### 3.1. Overview of the Biological Effects of Electric Fields

Recently, the electric fields have captured increasing spotlight as novel effectors in life processes [13]. It is well known that the tissues of the human body can generate electric fields and affect the body’s cells. On the one hand, naturally occurring electric fields can be found in many physiological processes, such as skin wound repair (40–200 mV/mm) and eye wound recovery (43.4–40.6 mV/mm) [14,15,16,17,18]. On the other hand, clinically applied electric fields on the human body are also emerging, including various types of medical devices such as cardiac pacemakers, medical imaging examinations, and wearable devices.

Electric fields consist of two categories: the alternating electric field and the direct current electric field. At present, studies on the biological effects of alternating electric fields have received much attention, such as the electrochemical treatment of tumors [19]. However, research on the biological effects of the dcEF is becoming a promising field. Here, by utilizing the RAW 264.7 cell line, a very commonly used the macrophage cell line in inflammation studies, we explored the effects of the dcEF on the transcriptomics of macrophages and their underlying signaling pathways.

### 3.2. Hub Genes

In this study, a total of 689 DEGs were obtained. Next, in order to focus on the influence of the electric field on individual genes, we selected eight differential genes with the largest change in expression from the up-regulated and down-regulated genes for subsequent analysis. A total of eight differential genes were selected: up-regulated *Fcgr1, Ypel3, Hcar2,* and *Mmp9* and down-regulated *Eid3, Lrp8, Ldlr,* and *Insig1* (Table 5 and Table 6).

*Fcgr1* is 2589 bp in length and is located on chromosome 3. As a protein-coding gene, *Fcgr1* encodes a protein called high-affinity immunoglobulin gamma Fc receptor I. Studies on the biological function of *Fcgr1* have mainly been focused on the signaling transduction in pain and inflammation processes. Recently, *Fcgr1* has been shown to play an indispensable role in the signal transduction of mouse inflammation and pain models, indicating that *Fcgr1* may be a potential therapeutic target for corresponding diseases [20,21]. In the present study, dcEF treatment increased the expression of *Fcgr1* in macrophages to 4.03-fold in the untreated group (Figure 4), suggesting that the *Fcgr1*-mediated signaling transduction for inflammatory pain may be involved in the signal response of macrophages to dcEFs.

*Ypel3* is located on chromosome 7 and has a length of 1057 bp. The protein it encodes is called yippee like 3, a growth-suppressive highly unstable protein. Current research suggests that *Ypel3* is a gene that inhibits tumor proliferation, suggesting that it may be a promising anti-cancer target protein [22,23]. In our data, dcEF exposure caused a rise in macrophage *Ypel3* expression levels to 4.84-fold, hinting that dcEF treatment may inhibit macrophage proliferation.

*Hcar2* is 1930 bp in length and is located on chromosome 5, and it encodes a protein called hydroxycarboxylic acid receptor 2. *Hcar2* is a novel negative regulator of macrophage activation and plays a critical role in host protection against pro-inflammatory attacks [24,26]. *Hcar2* is considered as a promising target may help to optimize multiple sclerosis (MS) therapies [25]. Here, dcEF exposure caused macrophage *Hcar2* levels to increase at least 5-fold, indicating that current stimulation may attenuate certain functions of macrophages.

*Mmp9* is located on chromosome 2 and has a length of 3175 bp. The encoded protein of *Mmp9* is matrix metallopeptidase 9. *Mmp9* is involved in a wide range of biological functions including macrophage differentiation, inflammation, bone metabolism, and tumor invasion [27,28,29]. In our study, electric field stimulation resulted in a 6.53-fold increase in macrophage *Mmp9*, suggesting that dcEF stimulation may affect macrophage differentiation and inflammatory responses.

*Eid3* is 1305 bp in length and is located on chromosome 10, and it encodes a protein called EP300 interacting with differentiation 3. At present, research on the function of *Eid3* is still rare, with one study showing that *Eid2* acts as a potent suppressor of nuclear receptor transcriptional activity [30]. Interestingly, our data showed that macrophage *Eid3* levels were reduced by at least 3-fold after dcEF stimulation, suggesting that electric field stimulation may inhibit the nuclear receptor transcription activity of macrophages.

*Lrp8* is located on chromosome 3 and has a length of 3291 bp, and it encodes a protein called low-density lipoprotein receptor-related protein 8. In recent years, *Lrp8* has been revealed to play a role in cell development and migration via the regulation of the canonical Wnt/β-catenin signaling pathway [33]. Hence, *Lrp8* is actively involved in the regulation of tumor and bone diseases [31,32]. In the present study, *Lrp8* levels were significantly decreased in RAW 264.7 macrophages after dcEF treatment, suggesting that electric field stimulation may affect macrophage development and migration.

*Ldlr* is 4549 bp in length and is located on chromosome 9, and it encodes the low-density lipoprotein receptor. As one of the key receptors for lipid metabolism in the body, *Ldlr* deeply regulates the metabolism of lipids and has become a promising drug target [34]. Our RNA-Seq data showed that *Ldlr* expression levels decreased significantly after dcEF exposure, suggesting that electric field stimulation may affect macrophage metabolism in lipids.

*Insig1* is located on chromosome 5 and has a length of 2667 bp, and it encodes a protein called insulin-induced gene 1. *Insig1* is primarily involved in macrophage-mediated innate immunity and cholesterol metabolism [35,36]. Interestingly, our data showed that the expression of macrophage *Insig1* was significantly decreased after dcEF stimulation, hinting that the electric field may inhibit the *Insig1*-mediated innate immunity of macrophages.

### 3.3. Sensitive lncRNAs

Surprisingly, in addition to DEGs, five dcEF-sensitive lncRNAs were also obtained (down-regulated: *AI480526, Gm26520, Gm26532, Gm28187,* and *Snhg20*) (Table 7).

*AI480526* is a 1697 bp lncRNA, and it is mainly present on chromosome 5 of mice [39]. Until now, studies have suggested that *AI480526* is related to the maturation of red blood cells [40,41]. However, according to our best knowledge, there have not yet been reports on the specific biological functions of *AI480526*. In the present study, the expression of *AI480526* in dcEF-treated macrophages was significantly reduced to 3-fold in the untreated group. Therefore, our data hinted that *AI480526* is related to the response of macrophages to dcEFs, which may provide clues for future biological function studies of *AI480526.*

*Gm26532* is a 690 bp lncRNA, and its specific biological function in mice is unknown. Interestingly, we found a homologous gene of *Gm26532* (gene id: 6607630) on *Drosophila sechellia* [42]. According to GO analysis, the gene functions on *Drosophila sechellia* are: guanyl-nucleotide exchange factor activity (molecular function), small GTPase (guanosine triphosphate enzyme) mediated signal transduction (biological process), and none (cellular component) [46]. These suggest that *Gm26532* may be an evolutionarily highly conserved gene that may possess critical biological functions. As shown in Figure 4, the expression level of *Gm26532* was decreased to two times less than that of the untreated group after dcEF treatment, indicating that *Gm26532* may be a response element for macrophage response to dcEF stimulation.

*Gm26520* is another lncRNA whose expression is reduced by more than 3-fold. It is mainly expressed on chromosome 12 and has a length of 1423 bp [39]. Till now, no reports of the biological function of *Gm26520* have been reported. Like *Gm26532, Gm26520* has a homologous gene in *Drosophila sechellia*. GO analysis showed that the gene functions of *Gm26520* on *Drosophila sechellia* are: glucosylceramidase activity (molecular function), sphingolipid metabolic process (biological process), and none (cellular component) [46].

*Gm28187* is mainly distributed on the first chromosome of mice and has a length of 2134 bp. In a mouse model of breast cancer tumors, *Gm28187* is upregulated by at least 2-fold in the tumor gland epithelium compared to the normal mammary gland tissue. Hence, *Gm28187* is considered to belong to the family of mammary tumor-associated RNAs (MaTARs) [43], which indicates that *Gm28187* should be involved in the migration and proliferation of tumor cells. In our study, exposure to dcEF reduced the expression of *Gm28187* in RAW 264.7 cells by 2-fold compared to the control group. Overall, *Gm28187* may be involved in the changes of macrophage migration and proliferation in response to dcEFs.

*Snhg20* is a 627 bp mouse lncRNA whose distribution is mainly located on chromosome 11. Unlike other lncRNAs, the biological functions of *Snhg20* have been intensively studied, and a unified conclusion has been reached. A large number of studies have demonstrated that the inhibition of *Snhg20* is closely related to the occurrence of tumors. Further molecular mechanism experiments have shown that *Snhg20* significantly promotes the proliferation of various tumor cells, indicating that *Snhg20* is a positive regulator of tumor cell proliferation. In our experiments, the expression of *Snhg20* in the dcEF exposure group was significantly decreased than that of the control group, suggesting that dcEF treatment may inhibit macrophages proliferation.

Hence, these above dcEF-sensitive lncRNAs provide meaningful clues for the further study of macrophage in response to electric field stimulation, and further exploration is needed.

### 3.4. Electrotaxis and Gene Expression 

A series of studies have been performed on the effect of dcEFs on cell gene expression. With the help of various devices, researchers have applied various types of current stimulation to cells, including the human adult dermal fibroblast cell line (HDF-a) (dcEF: 100 mV/mm, 1 h) [47], human adult epidermal keratinocytes (dcEF: 100 mV/mm, 1 h) [48], the human lung cancer cell line (CL1–5) (dcEF: 300 mV/mm, 2 h) [49], the human glioblastoma cell line (U87 mg), and the medulloblastoma cell line (DAOY) (dcEF: 250 mV/mm, 8 h) [50]. The results of gene expression profiling have shown that the expression characteristics of genes in cells are so diverse that we could not find a common gene that was regulated in all these studies. In our present study, the mouse macrophage cell line RAW 264.7 was stimulated with an electric field (dcEF: 200 mV/mm, 4 h). Interestingly the “Transcription” pathway, which is the only pathway that has been stimulated in all previous studies, was activated (Table 8). Therefore, we speculated that the transcription pathway may play an key role in cell’s electrotaxis.

### 3.5. Oxidative Stress and Cell Migration

The oxidative stress system is a key signal bridge for the directional movement of cells caused by external physical electric field stimulation. In 2013, electric field stimulation (dcEF: 200 mV/mm, 30–60 min) induced the directional migration of glioma cells (U251, 8,7 and C6) to the cathode and led to markedly intracellular ROS (Reactive Oxygen Species) production. Meanwhile, glioma cells significantly produce hydrogen peroxide and superoxide by up to 2–3-fold more than untreated cells. Moreover, with the help of genetics and pharmacology of the ROS system, the researchers found that the generation of superoxide, but not hydrogen peroxide, would be an indispensable link in the dcEF-induced directional migration of glioma cells [51]. In 2015, by using a new microfluidics chip system, researchers found that a dcEF (100–400 mV/mm, 2 h) caused NIH 3T3 fibroblast migration and that cellular ROS production was EF-strength-dependent [52].

Consistent with these findings, in our study, the application of a dcEF (200 mV/mm, 4 h) caused a significant change in the gene expression profile of RAW 264.7 cells, and an enrichment analysis of DEGs showed that the oxidation stress pathway was markedly activated. Hence, previous studies and our findings further show that the oxidative stress system is one ‘‘bridge’’ coupling the physical electric field stimulation to the intracellular signals during dcEF-mediated cell directional migration.

### 3.6. Macrophages Electroporation Activation

In order to maximize transfection efficiency and avoid unnecessary macrophage activation, electroporation is widely used to transfect foreign genes into macrophages [53]. It is worth noting that when an electroporation experiment is performed, the commonly used voltage strength (10,000–100,000 mV/mm) is more than two orders of magnitude (100–7500 mV/mm) higher than the electric field strength for investigating the biological effects of electric fields. Recently, when the bovine NRAMP1 gene was transfected into RAW 264.7 cells using electroporation, the applied electric field intensity was as high as 75,000 mV/mm [54].

The effect of the electric field on the cells varies with the strength and duration of the applied electric field. First of all, in terms of electric field stimulation intensity: When a strong-intensity electric field (75,000 mV/mm) is applied (electroporation), the cell membrane easily forms small pores, which facilitates the entry of foreign DNA into the cell. In contrast, when the electric field strength is weak (10–100 mV/mm), a series of responses occurred in the cell, including electrotaxis, gene expression profile changes, and protein structure rearrangement. Secondly, in terms of the duration of the electric field stimulation. The duration of electroporation is usually very short (ranging from a few microseconds to a few milliseconds), which helps cells to survive high-intensity electric field stimulation. In contrast, the duration of ordinary electric field strength is longer than electroporation (1–8 h), and cells undergo gene expression, electrotaxis, and protein structure changes during this process. Collectively, the effect of electric fields on cells is affected by a variety of factors, which deserves further study.

### 3.7. Electric Field Strength

#### 3.7.1. Experimental Study

In the process of studying the effects of physical electric fields on cells, the choice of electric field experimental intensity and duration is a step worthy of attention. In 2013, glioma cells (U251, 87, and C6) were stimulated by an electric field (dcEF: 200 mV/mm, 30–60 min) to study the intracellular signal mechanism of superoxide in dcEF-mediated cell directional migration [51]. In 2015, a dcEF (100–400 mV/mm, 2 h) was applied to NIH 3T3 fibroblasts in order to investigate the correlation between ROS production and EF-induced cell migration [52]. In addition, the human lung cancer cell line (CL1–5) (dcEF: 300 mV/mm, 2 h) [49], the human glioblastoma cell line (U87 mg), and the medulloblastoma cell line (DAOY) (dcEF: 250 mV/mm, 8 h) were used to study the effect of electric field stimulation on cellular gene expression [50].

In the field of macrophages, human monocyte-derived macrophages (dcEF: 5–300 mV/mm, 2 h) [9], mouse bone marrow-derived macrophages (dcEF: 300 mV/mm, 4 h) [11], mouse peritoneal macrophages (PMs) and bone marrow-derived macrophages (BMDMs) (dcEF: 400 mV/mm, 3 h) [12] have been used to study various biological effects of electric fields on macrophages. The interpretation of the above results suggests that the proper stimulation condition of an electric field on cells is in the range of 100–400 mV/mm (0.5–8 h). Taken together, in this study, electric field stimulation (dcEF: 200 mV/mm, 4 h) was selected to stimulate RAW 264.7 cells for subsequent studies. 

#### 3.7.2. Molecular Dynamics Study

In the field of using molecular dynamics to study the structural changes of proteins under an electric field, it is considered that an electric field intensity greater than 5 × 10^5^ mV/mm will cause significant protein structural changes, while a moderate physical electric field will have remarkable impacts on protein dynamics [55,56,57,58]. In a 10 ns molecular dynamics simulation research system, tubulin protein was applied with external fields (7.5 × 10^8^ mV/mm), and the results showed that the electric field induced obvious conformational rearrangements in an electric field strength-dependent way [59]. In another molecular dynamics simulation study, the soybean hydrophobic protein was stimulated with an electric field of 2000–4000 mV/mm, and the results demonstrated that the electric field stimulation at this intensity had no effect on the structure of the protein. Meanwhile, a higher electric field intensity (3 × 10^6^ mV/mm) can significantly affect the conformation and surface structure of the soybean hydrophobic protein [60]. In 2017, by using molecular dynamics methods, researchers found that the structure of photoproteins under electrical field stimulation (2 × 10^5^–5 × 10^5^ mV/mm) were significantly changed [61].

In our molecular dynamics system, in order to maintain consistency with cell electric field stimulation experiments (200 mV/mm), the electric field strength was selected to cover the achievable range of 10–500 mV/mm (10, 50, and 500 mV/mm). Unexpectedly, at 500 mV/mm, the structure of the protein underwent major changes, and even its normal secondary structure could not be maintained. As for this difference, we have not yet found a more specific reason, presumably related to different cell types, so it may need further investigation.

### 3.8. Electric Field-Induced Changes in Protein Structure

In this study, based on the results of molecular dynamics simulations, the protein structure was changed under the action of electric fields of different intensities (Figure 7, Figures S5 and S6). Given that the main effect found in the molecular dynamics calculations was that increasing electric fields seemed to provoke a significant structural disorder in protein, it is a noteworthy question to discuss the effect of electric fields on protein structure.

In our opinion, the following factors may have been responsible for this phenomenon: 

(1) The structural characteristics of the protein was the basis for the electric field-caused protein structure changes. On the one hand, proteins were loaded with both positive charges (amino terminus) and negative charges (carboxy terminus), and the charged groups carried by the side chain of the protein were also affected by the electric field. On the other hand, under the electric field environment, the main forces that maintain the protein structure may have been significantly changed, including van der Waals forces, hydrophobic interactions, and ionic bonds. (2) Electric field-caused protein structure changes were closely related to the strength of the electric field. As shown in Figure 7, the lower electric field strength (10 mV/mm) had the smallest effect on the protein structure, while the medium electric field strength (50 mV/mm) had a more significant effect on the structure of some proteins. It is worth noting that under the effect of high-intensity electric field strength (500 mV/mm), the structure of most proteins was completely disrupted.

Additionally, we believe that the mechanism of the effect of electric field on protein structure can be explored in depth from the following aspects:

(1) Experimental verification: A variety of experimental methods can be used to study the effects of electric fields of different intensities on protein structure, such as testing CD (Circular Dichroism) spectroscopy to investigate changes in secondary protein structure, using genetic engineering to mutate key residues to verify the effects of electric field on single amino acid residues, and detecting the influence of the electric field on protein-related signaling proteins to explore the signal pathways that may be involved. (2) Molecular dynamics simulation: Theoretically, all electric field strengths (at least in our experimental system, from 10–500 mV/mm) can be used to simulate the changes in the structure of each hub protein under the electric field. Unfortunately, due to limited experimental resources and conditions, we could not complete the above work in this study, so it will be the direction of our future research.

Collectively, by using molecular dynamics methods, we obtained the following findings: (1) Increasing electric fields seemed to provoke a significant structural disorder of protein structure. (2) In terms of the amplitude of electric field stimulation, the effect of 10 mV/mm electric field exposure on the protein was very slight, while 500 mV/mm electric field exposure made the protein structure completely disordered.

It is worth questioning what way electric field exposure causes changes in protein structure? Is this transition smooth or does some discontinuity appear at some threshold field value? These are issues that need to be resolved in the future.

### 3.9. Voltage-Gated Channels 

The voltage-gated calcium channel protein was a bridge in which the electric fields affected cells. Pulsed electric fields (PEFs) directly affect the voltage-gated ion channels of U87 glioblastoma cells, which can be blocked by pharmacological antagonists of ion channels. Moreover, the electric field-induced cell membrane depolarization effect can also be blocked by pharmacological antagonists of certain cationic channels [62]. Therefore, these findings suggest that voltage-gated ion channels may be one of the bridges where electric fields affect cells. Moreover, voltage-gated calcium channels are also considered to be one of the main ways that electromagnetic fields (EMFs) affect cells [63]. In addition, the results of molecular dynamics studies have shown that these four phenylalanine residues at the internal gate region are very important for the activation of human voltage-gated calcium channels [64].

### 3.10. Resting Potential

Membrane potential is an important basis for cells to perform various physiological functions. In general, there is a significant difference in the ion concentration on the inside and outside of the cell membrane, which gives rise to an electrical potential difference across cell membranes is called the membrane potential. As an important property of many cells, changes in resting membrane potential affect numerous biological processes, including energy metabolism, muscle contraction, neural networks, and macrophage polarization [65,66].

Recent studies have shown that resting membrane potential can serve as a breakthrough for regulating macrophage polarization. In the THP-1 macrophage model, pharmacological antagonists targeting ATP-sensitive potassium channels reduce M1 marker secretion and gene expression, and this effect can be reversed by corresponding ATP-sensitive potassium channel agonists. Moreover, this ATPase-sensitive potassium channel antagonist enhances the expression of certain M2 markers during the M2 polarization of macrophages [67]. This shows that the ultimate goal of regulating macrophage polarization can be achieved by controlling the resting membrane potential of macrophages.

Due to the limitations of our experimental conditions, the resting potential of dcEF-treated RAW 264.7 cells could not be obtained in our study. Previous literature has shown that the resting membrane potential of RAW 264.7 macrophages is approximately −55 mV [68]. At present, with the in-depth study of the regulation of macrophages by electric fields, a question gradually arises: by what means do electric fields regulate the activity of macrophages (such as polarization)? Resting membrane potential may be one of the answers. Unfortunately, to the best of our knowledge, no direct research on the electric field to regulate the resting membrane potential of macrophages has been done. We speculate that this is worthy of further research, and it may provide a meaningful exploration for revealing the molecular mechanism of electric field regulation of macrophage activity.

## 4. Materials and Methods

### 4.1. The Outline of This Work

Briefly, this study mainly included the following three parts:

Firstly, the electric field-sensitive genes were obtained from RAW 264.7 macrophages. RAW 264.7 cells were exposed to a direct electric field of 200 mV/mm for four hours and were subjected to RNA-Seq analysis. After analyzed by a series of bioinformatics tools, 689 differential genes were acquired. After GO, KEGG, and PPI analyses, the cellular signaling pathways composed of these differential genes were explored. 

Secondly, 8 DEGs with the greatest variation at the transcriptional level were selected for subsequent analysis. The three-dimensional protein structure of these DEG genes was constructed. By using Modeller 9.21 and Rosetta 3.9, a three-dimensional structural model for each DEG protein was originally established. Subsequently, a 200 ns molecular dynamics simulation for each DEG protein was carried out by using the GROMACS 2018.2 software package, and the protein structures optimized with molecular dynamics simulation were finally acquired.

**Figure 8:**
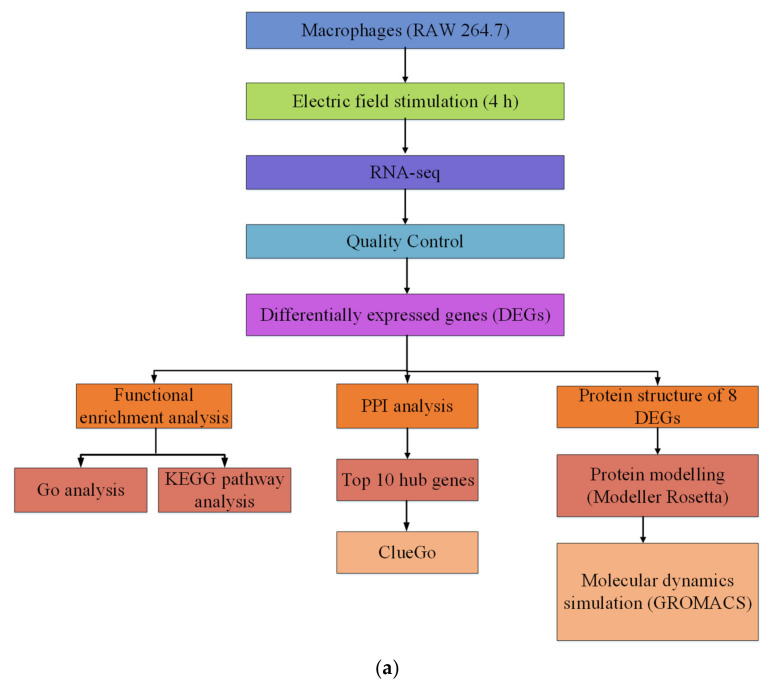

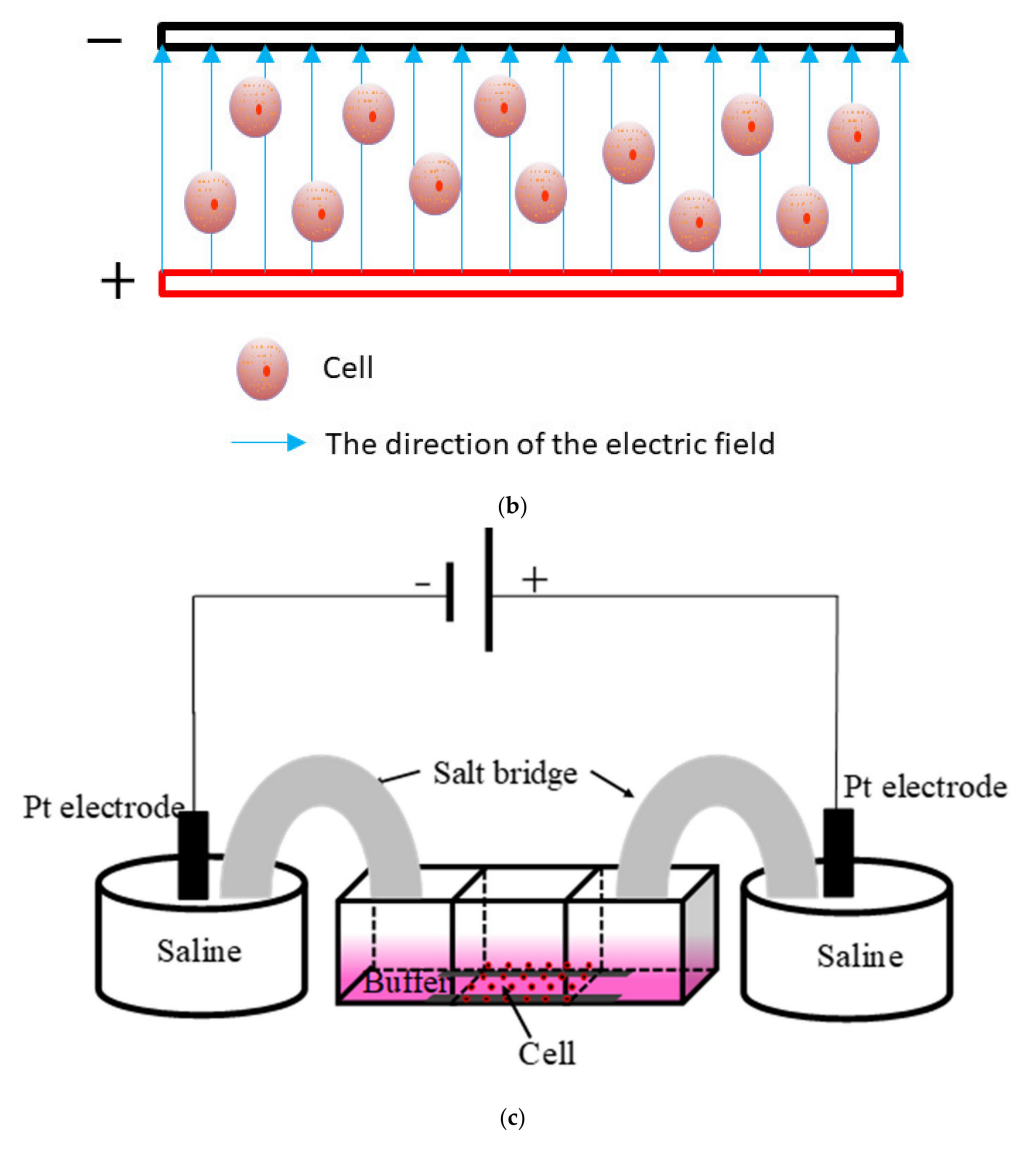

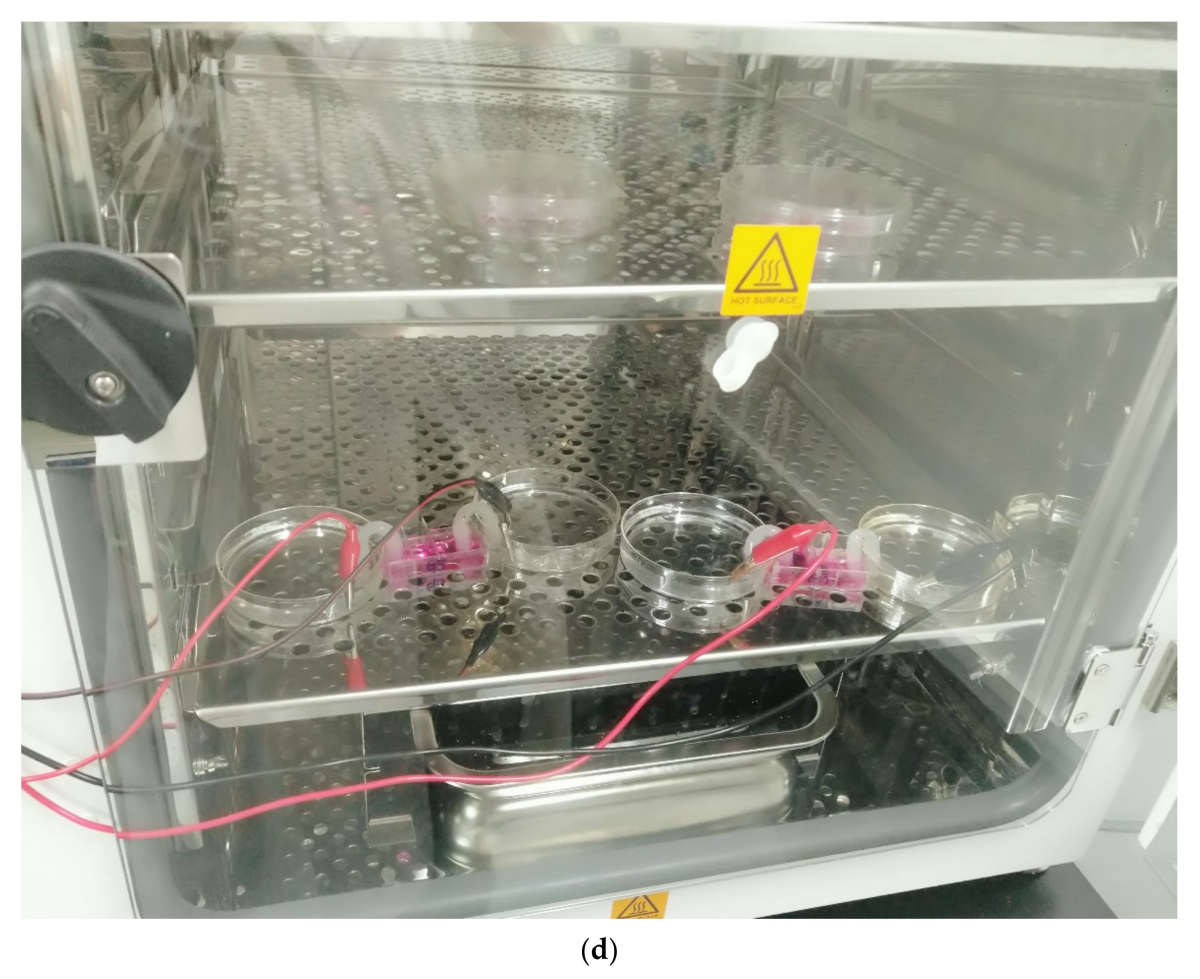
Schematic diagram. (**a**) Study workflow. Red object: cell; blue arrow: direction of electric field. (**b**) The schematic diagram of the movement of cells in the electric field. (**c**) The structure of the chamber. (**d**) The electric field chamber in the incubator.

Finally, molecular dynamics (at least 200 ns) were used to simulate the molecular motion of the DEG proteins at three electric field strengths (10, 50, and 500 mV/mm), and a series of molecular motion characteristics, such as the RMSD, RMSF, and R*g* of the protein were obtained (Figure 8a). As shown in Figure 8b, the cells were cultured in a chamber, and an electric field was applied. 

### 4.2. Materials

Dulbecco’s modified Eagle’s medium (DMEM), fetal bovine serum (FBS), a trypsin–EDTA (ethylenediaminetetraacetic acid) solution (0.05% Trypsin–EDTA), β-mercaptoethanol, TRIzol, and a Qubit^®^ RNA Assay Kit were acquired from Gibco™ and Invitrogen™ (Thermo Fisher Scientific, Inc., Waltham, MA, USA). Penicillin (10,000 units/mL)/streptomycin (10,000 μg/mL) antibiotics and trypsin were obtained from Merck-Millipore. Cell culture dishes were purchased from Corning, Inc. (Corning, NY, USA). The SYBR^®^ Premix Ex Taq and PrimeScript ^TM^ 1st Strand complementary DNA (cDNA) Synthesis Kit were from TaKaRa Biotechnology (Dalian, China). M-MuLV Reverse Transcriptase (RNase H-), USER Enzyme, Phusion High-Fidelity DNA polymerase, and NEBNext^®^ UltraTM RNA Library Prep Kit was from Illumina^®^ (NEB, Ipswich, MA, USA).

### 4.3. Cell Culture

RAW 264.7 cells were obtained from the Stem Cell Bank of the Chinese Academy of Sciences (Shanghai, China). Cells were cultured in a DMEM medium supplemented with 10% fetal bovine serum (heat-inactivated) and 1% penicillin/streptomycin antibiotics (100×). Cells were maintained at 37 °C with 5% CO_2_ in a fully humidified air incubator, and the culture medium was refreshed every 2–3 days.

### 4.4. Electric Field Stimulation

#### 4.4.1. Construction of Electrotaxis Chambers

The specific structure of the chamber is as follows. The chamber was a rectangular structure bonded with glass bonding, with 5 × 2 × 2 cm (length × height × width) (the thickness of the glass was 1 cm). The glass slides with 2 × 0.5 × 0.15 cm (length × width × thickness) were bonded on both sides in the middle of the bottom. In addition, glasses with 2 × 1cm (length × width) were bonded in the middle of the bottom of the chamber with a spacing of 1 cm. Then, a narrow passage was formed at the bottom (Figure 8c).

The electric field apparatus contained a self-made chamber that supplied an electric field across a salt bridge. To make the agar salt bridge, 4 wt.% agar was added to the saturated KCl solution; then the mixture was boiled until the agar was dissolved and poured into the custom U-shaped glass tube [69]. The system of this electric chamber was as follows: the positive and negative leads of the DC power supply (UNI-T/UTP-3305) were connected with the self-made conductive platinum (Pt) wire. Then, the positive and negative conductive Pt wires were placed in a 0.9% NaCl conductive solution, and the positive and negative conductive solutions were connected with both sides of the EF chamber by the salt bridge to form a conductive circuit. During the experiment, the target voltage at both sides of the middle chamber was 2 V. Actually, the actual voltage of the external device providing electric field was 10 V. The electric field strength (200 mV/mm) was determined by measuring the voltage between two salt bridges. After the experiment started, a multimeter was used to check the voltage value at both sides of the electric field chamber every 30 min to ensure that the system’s voltage was kept stable (Figure 8d).

#### 4.4.2. Preparation of the Chamber

Each chamber was washed 3 times with sterile phosphate buffer saline (PBS) for 5 min each. Subsequently, each chamber was placed in a petri dish (100 mm in diameter), the lid of the petri dish was opened, and the petri dish was placed in a clean bench for ultraviolet light irradiation. After 1 h, the petri dish was covered and placed in this clean bench.

#### 4.4.3. Preparation of Cells

Next, an experiment of seeding cells into a chamber was performed. RAW 264.7 cells were routinely cultured in cell culture dishes (60 mm diameter). Approximately 70–80% of adherent cells were used for cell seeding experiments. Before the experiment, the cell morphology, which should have been oval, was observed under a light microscope. The cells were gently washed twice with PBS (37 °C) to remove residual cell debris. Subsequently, the cells in the dish were gently rinsed with a complete medium (DMEM medium supplemented with 10% FBS and 1% penicillin/streptomycin antibiotics (100×)) until the cells were completely detached. After centrifugation at 800 rpm/min for 5 min, the cell pellet was resuspended at the bottom of the centrifuge tube with 1 mL of complete medium. After the cells were counted, 2 mL of cell suspension (375,000/chamber) were seeded into the chamber. After overnight, the morphology of the cells in the chamber, which should have been oval, was observed.

#### 4.4.4. Application of Electric Field

After the electric field stimulation device was set up, the chamber was connected to the electric field stimulation system (as shown in Figure 8). After the cells were stimulated with a dcEF (200 mV/mm) for 4 h, the cells were subjected to various tests.

For the cell samples to be sequenced, cells were rinsed for three times with PBS, and they were fully lysed by the addition of 1 mL of TRIzol into the chamber. Subsequently, samples were immediately frozen with liquid nitrogen and stored in liquid nitrogen. Finally, the sequencing was performed by Novogene Co Ltd. (Bejing, China). 

Images were obtained using A-Plan 10 Χ/0.25 PH1 and long-distance A-Plan 5 Χ PH0 objectives on a microscope (DM IRB, Leica Microsystems GmbH, Wetzlar, Germany) with a Leica camera (DFC 420C, Leica Microsystems GmbH, Wetzlar, Germany). Images were acquired with software (LAS v4.0, Leica Microsystems GmbH, Wetzlar, Germany).

In our experimental system, the Joule heating generated by electric field stimulation could be ignored for the following reasons: (1) The current produced by this experimental device was very small (just a few microamperes), so the Joule heat generated by the system was also very small, which could also be found in other similar devices [70]; (2) cells were stimulated by electric current in the incubator, where the excess heat was dispersed by the automatic control system by adjusting the ambient temperature.

### 4.5. Gene Expression Analysis

This method has been previously described [71]. In brief, the total RNA was extracted from cells by using the TRIzol under the manufacturer’s instruction. cDNA was transcribed from 1 μg of RNA by using the PrimeScript ^TM^ 1st Strand cDNA Synthesis Kit. The gene expression was detected with the SYBR^®^ Premix Ex Taq system using the MX3000P Real-Time PCR System (Stratagene). 

Real-time PCR procedures were generally as follows with minor modifications: 94 °C for 30 s, 95 °C for 5 s, 58 °C for 30 s, and 72 °C for 1 min for 32 cycles. Data were normalized to GAPDH (glyceraldehyde-3-phosphate dehydrogenase) levels using the comparative 2^−ΔΔCT^ m ethod. Primers for genes are listed in Table 9. The gene expression data were conducted in duplicates and repeated three times.

### 4.6. RNA-Seq Sample Collection and Preparation

#### 4.6.1. RNA Quantification and Qualification

RNA contamination and degradation were detected on 1% agarose gels. RNA purity was examined by using a NanoPhotometer^®^ spectrophotometer (IMPLEN, Westlake Village, CA, USA). RNA integrity was investigated with an RNA Nano 6000 Assay Kit of the Bioanalyzer 2100 system (Agilent Technologies, Santa Clara, CA, USA). RNA concentration was determined by using Qubit^®^ RNA Assay Kit in Qubit^®^2.0 Fluorometer (Life Technologies, Carlsbad, CA, USA). 

#### 4.6.2. Library Preparation for Transcriptome Sequencing

A total amount of 3 µg RNA per sample was extracted as input material for the RNA sample preparations. Sequencing libraries were constructed using the NEBNext^®^ UltraTM RNA Library Prep Kit for Illumina^®^ (NEB, Ipswich, MA, USA) according to the manufacturer’s protocols.

In brief, mRNA was isolated from total RNA with the poly-T oligo-attached magnetic beads. Fragmentation was performed by using divalent cations under elevated temperature in a NEBNext First Strand Synthesis Reaction Buffer (5×). First-strand cDNA was synthesized with the M-MuLV Reverse Transcriptase (RNase H-) and a random hexamer primer. Second strand cDNA synthesis was subsequently carried out using RNase H and DNA Polymerase I. Remaining overhangs were converted into blunt ends with polymerase/exonuclease functions. After 3′ ends of DNA fragments were adenylated, a NEBNext Adaptor with hairpin loop structure was ligated to prepare for subsequent hybridization. Next, in order to acquire cDNA fragments of preferentially 250~300 bp in length, the library fragments were purified by using AMPure XP system (Beckman Coulter, Beverly, USA). Subsequently, 3 µl of USER Enzyme (NEB, USA) was utilized with size-selected, adaptor-ligated cDNA at 37 °C for 15 min, followed by 5 min at 95 °C before PCR. Then, PCR was carried out by using Universal PCR primers, Phusion High-Fidelity DNA polymerase, and index (X) primers. Finally, PCR products were purified (AMPure XP system), and library quality was examined with the Agilent Bioanalyzer 2100 system.

#### 4.6.3. Clustering and Sequencing

The clustering of the index-coded samples was conducted by the cBot Cluster Generation System using the TruSeq PE Cluster Kit v3-cBot-HS (Illumia). After cluster generation, the library preparations were sequenced with the Illumina Hiseq platform which finally obtained 125 bp/150 bp paired-end reads.

### 4.7. RNA-Seq Data Analysis

#### 4.7.1. Quality Control

Raw data (raw reads) in fastq format were firstly processed through in-house Perl scripts. In this step, clean data (clean reads) were acquired by removing low quality reads from raw data, including reads containing adapter and ploy-N. Meanwhile, the Q20, Q30, and GC content of the clean data were calculated. All the downstream analyses were based on the clean data with high quality.

#### 4.7.2. Reads Mapping to the Reference Genome

Reference genome and gene model annotation files were directly retrieved from the genome website. Index of the reference genome was constructed using Hisat2 v2.0.5, and paired-end clean reads were aligned to the reference genome using Hisat2 v2.0.5. The Hisat2 was selected as the mapping tool.

#### 4.7.3. Quantification of Gene Expression Level

The FeatureCounts v1.5.0-p3 was used to count the read numbers mapped to each gene. Then, the FPKM (fragments per kilobase of exon model per million reads mapped) of each gene was calculated based on the length of the gene and read count was mapped to this gene. 

#### 4.7.4. Differential Expression Analysis

Differential expression analysis was conducted by using the DESeq2 R package (1.16.1). DESeq2 provided statistical routines for determining differential expression in digital gene expression data using a model based on the negative binomial distribution. The resulting *p*-values were adjusted using the Benjamini and Hochberg’s approach to control the false discovery rate. Genes with an adjusted *p*-value < 0.05 found by DESeq2 were considered as differentially expressed.

#### 4.7.5. GO and KEGG Enrichment Analysis of Differentially Expressed Genes

The GO enrichment analysis of differentially expressed genes was analyzed by the ClusterProfiler R package [72], in which gene length bias was adjusted. GO terms with adjusted *p* value less than 0.05 were considered significantly enriched by differential expressed genes.

To interpret the potential mechanism and pathways associated with DEGs, the enrichment analysis was performed based on Metascape online tool (http://metascape.org/) [73], and the significant biological processes were identified by using the gene lists. Metascape is an online tool for gene annotation analysis that queries a great number of databases including GO functional, KEGG pathways, and Hallmark Gene Sets.

To further understand the functions and pathways of most abundantly expressed genes, DEGs were subjected to GO classification and functional analysis using PANTHER (http://www.pantherdb.org/) [74]. According to the PANTHER GO classification method, three main GO categories include MF, BP, and CC were displayed.

KEGG is a database resource for understanding high-level functions of the biological field, including the cell, the ecosystem, and the organism (http://www.genome.jp/kegg/). Here, we employed the ClusterProfiler R package to test the statistical enrichment of differential expression genes in KEGG pathways. In addition, in order to clearly present the KEGG pathway maps, a Cytoscape plug-in (KEGGParser) was used to investigate and optimize biological networks.

#### 4.7.6. Integration of Protein–Protein Interaction (PPI) Network and Module Analysis

The STRING database (http://string-db.org) aims to provide a comprehensive assessment and integration of PPIs, including indirect (functional) and direct (physical) associations [75]. STRING (version 11.0) covers 24,584,628 proteins from 5090 organisms (4445 bacteria, 477 eukaryotes, and 168 archaea) and 3,123,056,667 total interactions. In order to assess the interactive relationship among DEGs, the DEGs were firstly mapped to STRING. Only experimentally validated interactions with a combined score >0.4 were considered as significant. Subsequently, the PPI network was built by using the Cytoscape software (Ver. 3.7.1). The plug-in Molecular Complex Detection (MCODE) was employed to screen the modules of PPI network in Cytoscape. Moreover, an enrichment study was carried out for DEGs of the modules, and *p* < 0.05 was selected to have a striking difference.

To further investigate the pathways of the identified DEGs, pathway enrichment was analyzed by Cytoscape software with the clueGO and Cluepedia plug-ins. The ClueGO plug-in (http://apps.cytoscape.org/apps/cluego) can detect the molecular mechanisms of large gene lists by identifying significant GO and KEGG terms pathways [76]. The Cluepedia plug-in (http://apps.cytoscape.org/apps/cluepedia) has an intuitive and expressive visualization, which is capable of searching pathway-associated markers [77]. In our work, a KEGG pathway enrichment study was conducted by using the ClueGO and CluePedia tool kits, and a *p* value of <0.05 and a kappa coefficient of 0.4 were selected as threshold values.

In addition, CytoHubba, another plug-in program, was used to classify the top-ranked hubs in the network of the significant identified genes. A total of 10 hub genes were identified based on a series of approaches including MCC (Maximal Clique Centrality), DMNC (Neighborhood Component), MNC (Maximum Neighborhood Component), Degree, EPC (Edge Percolated Component), BottleNeck, EcCentricity, closeness, radiality, betweenness, stress, and clustering coefficient [78,79].

### 4.8. Protein Modeling

Protein sequences were retrieved from the NCBI nucleotide database (https://www.ncbi.nlm.nih.gov/protein/), and the BLAST module was employed to align the protein sequences with the PDB database [80]. For each protein, three templates (query cover > 30%) were chosen as templates for following homology modeling (Table 3). Subsequently, three-dimensional homology models of DEG proteins were constructed using Modeller (9v21) [81]. Multiple-template modeling approaches, including Salign, Align2d, and Model modules were used in the modeling process. For each protein, 1000 candidate models were generated, and the best model was acquired based on scores calculated from DOPE. The protein template was from the RCSB (Research Collaboratory for Structural Bioinformatics) PDB Protein Data Bank (PDB IDs see Table 3)

### 4.9. De Novo Modeling of Proteins

Because the BLAST results of several proteins (Eid3, Insig1, and Ypel3) from NCBI (query cover <30%) was too low to generate protein models by using Modeller (9v21), we employed the ROSETTA3.9 (de novo modeling script) [82] to generate three-dimensional protein models for these proteins. For each protein, 1000 candidate models are generated and the one with the lowest score was selected as the best theoretical protein model.

### 4.10. Molecular Dynamics Simulations

The MD simulation of the DEG proteins was carried out by using the GROMACS2018.2 package [83] in the Linux environment, and all simulations were run with the CHARMM36 force field [84].

#### 4.10.1. Molecular Dynamic Simulation: Protein in Water

Molecular dynamics simulations for proteins were conducted in similar conditions with various minor modifications. The protein was fully solvated in an octahedron box with SPC (simple point charge) water molecules (1.0 nm). The system was neutralized by adding Na^+^ or Cl^-^ ions, and periodic boundary conditions were utilized in all directions. The energy minimization of the protein was performed with 50,000 steps of steepest descent with the max force of less than 100 KJ/mol. Then, the system was set to the equilibration phases using NVT (constant number (N), volume (V), and temperature (T)) (50 ps, 300 K) and NPT (constant number (N), pressure (P), and temperature (T)) (100 ps, 300 K, 1.0 bar). Molecular dynamics simulation was performed for 200 ns for each protein. 

#### 4.10.2. Molecular Dynamic Simulation: Protein Under dcEF

For the molecular dynamics simulation of proteins under a dcEF, at least 200 ns was simulated in the NPT stage. When the simulation conditions were set, the electric field limiting conditions (electric-field-x = 10, 50, and 500 mV/mm) were added to the md.mdp file (Supplementary File 5), with other conditions remained the same as “protein in water.”

#### 4.10.3. Molecular Dynamic Simulation Analysis

The MD trajectory obtained after MD simulation was analyzed using GROMACS utilities to produce the RMSD, RMSF, and radius of gyration. The Xmgrace tool was used to produce various plots. Ramachandran plot analysis was carried out with PROCHECK Ramachandran plots [85] (http://www.ebi.ac.uk/thornton-srv/databases/pdbsum/Generate.html). The three-dimensional protein structure and MD simulation movie of protein models were generated by Pymol software (Delano, W.L. The Pymol Molecular Graphics System (2002) DeLano Scientific, SanCarlos, CA, USA. http://www.pymol.org).

## 5. Conclusions

In summary, our work provided helpful data for exploring dcEF-sensitive hub proteins of macrophages, and it explored the signaling pathway of macrophages in response to electric field-like physical stimuli. Based on our findings, we also speculated that the transcription pathway may play an key role in cell’s electrotaxis.

## Figures and Tables

**Table 1:** Details of Long non-coding RNA (lncRNA) and MicroRNA (miRNA).

Gene_Biotype	Gene_Name	log2FoldChange	*p*-Value	Padj
lncRNA	*Gm26532*	−2.470287104	1.61 × 10^−13^	4.04 × 10^−10^
	*Gm28187*	−2.088392064	9.78 × 10^−12^	1.85 × 10^−8^
	*AI480526*	−3.034810761	1.58 × 10^−9^	1.71 × 10^−6^
	*Gm26520*	−3.026474908	5.02 × 10^−9^	4.46 × 10^−6^
	*Snhg20*	−1.323550221	1.30 × 10^−8^	7.69 × 10^−6^
	*miR17hg*	−2.836327803	1.36 × 10^−7^	4.89 × 10^−5^
	*Gm46224*	−1.74087554	1.06 × 10^−6^	0.000208401
	*Gm12708*	2.480747891	4.30 × 10^−6^	0.000612157
	*Gm40723*	1.061737181	1.69 × 10^−5^	0.001775738
	*CT030636.2*	−2.393353924	2.72 × 10^−5^	0.002486353
	*Gm47015*	−1.934651075	3.57 × 10^−5^	0.003014666
	*Gm16755*	−2.461324991	5.86 × 10^−5^	0.004243817
	*miR142hg*	−1.680214964	7.70 × 10^−5^	0.005032238
	*Gm26890*	−2.201929478	0.000168331	0.008675651
	*Gm7292*	−1.230352255	0.000211049	0.010247751
	*Lsmem2*	−2.439159554	0.000218217	0.010395258
	*Gm38399*	−1.345909825	0.000265851	0.011965918
	*Gm26792*	−1.806478371	0.00035872	0.014640637
	*Gm17300*	−2.092545053	0.000750651	0.023785141
	*Gm36738*	1.207513949	0.001022396	0.029021064
	*Gm47343*	1.203380022	0.001192886	0.031492603
	*Gm17745*	1.042574015	0.001198205	0.031522819
	*Pldi*	−2.920635148	0.001270895	0.032583666
	*Gm38102*	−2.486096408	0.001308696	0.033258798
	*Gm26711*	−2.19928712	0.001578787	0.036963198
	*Gm21781*	−1.366279119	0.001838241	0.040183135
	*Gm26869*	−2.327597738	0.001839189	0.040183135
	*Gm35037*	−1.260055171	0.001948831	0.041708195
	*Gdap10*	−1.315142974	0.002101718	0.043726474
	*Gm21817*	−2.842246271	0.002255472	0.046214222
miRNA	*Gm23935*	9.74 × 10^−7^	0.000196164	Gm23935
	*Gm24270*	2.73 × 10^−6^	0.000453662	Gm24270
	*miR142b*	3.27 × 10^−6^	0.000523127	miR 142b
	*miR 23a*	4.69 × 10^−5^	0.003747572	miR 23a
	*miR 365-2*	5.96 × 10^−5^	0.004258302	miR 365-2
	*miR 23b*	0.000355791	0.014599983	miR 23b
	*miR 27a*	0.000605588	0.021022951	miR 27a
	*miR 221*	0.000608768	0.021077429	miR 221
	*miR 7-1*	0.000680236	0.022379617	miR 7-1

**Table 2:** Kyoto Encyclopedia of Genes and Genomes pathway (KEGG) pathway analysis of up-regulated and down-regulated DEGs.

Expression	Pathway ID	Name	Gene Count	%	Genes
Down-regulated	mmu01100	Metabolic pathways	32	15.69	*Gldc, Cbr3, Fdps, Pgd, Acat2, Cox17, Cyp51, Dpm1, Fasn, Fpgs, Gclc, Gclm, Gsr, Hmgcr, Hsd17b7, Lss, Nqo1, Pfkfb1, Pfkfb2, Piga, Sqle, Hmgcs1, Pank3, Mars2, Mgat2, Mat2a, Pfas, Impad1, Idi1, Vkorc1l1, Gbe1, Dhcr24*
	mmu05200	Pathways in cancer	7	3.43	*Fzd5, Igf1, Jag1, Nqo1, Skp2, Lpar5, Txnrd1*
	mmu04152	AMP-activated protein kinase (AMPK) signaling pathway	6	2.94	*Prkab2, Fasn, Hmgcr, Igf1, Pfkfb1, Pfkfb2*
	mmu00100	Steroid biosynthesis	6	2.94	*Cyp51, Hsd17b7, Lss, Sqle, Soat2, Dhcr24*
	mmu00900	Terpenoid backbone biosynthesis	6	2.94	*Fdps, Acat2, Hmgcr, Hmgcs1, Idi1, Pdss1*
Up-regulated	mmu01100	Metabolic pathways	49	24.02	*Gstt3, Tecr, Cth, Gpt2, Acy1, Acadm, Acaa1a, Prdx6, Ass1, Akr1b7, Car6, Comt, Cox6a2, Dgka, Galc, Hagh, Gpx1, Mthfd2, Nos2, Pla2g4a, Dhrs3, St6gal1, St3gal3, Cox7a2l, Pycr1, Mars, Uros, Smox, Gatb, Pigv, Mpst, Tpk1, Pigl, St3gal6, Extl1, Aldh18a1, Dhodh, Sqor, Txndc12, Ntpcr, G6pc3, Coasy, Amdhd1, Pcbd2, Pck2, Grhpr, Rdh12, Acsbg1, C1galt1*
	mmu05200	Pathways in cancer	17	8.33	*Gstt3, Bad, Gadd45a, Gnb5, Gng5, Gngt2, Il15, Il2rg, Jak2, Bbc3, Mmp9, Nfkb2, Nos2, Ralgds, Traf1, Mapk3*
	mmu04151	PI3K-Akt (Phosphatidylinositol 3-kinase (PI3K)/protein kinase B (AKT)) signaling pathway	12	5.88	*Bad, Gnb5, Gng5, Gngt2, Magi1, Il2rg, Itgb5, Jak2, Tlr2, Mapk3, G6pc3, Pck2*
	mmu05202	Transcriptional misregulation in cancer	10	4.9	*Gadd45a, Ddit3, Fcgr1, Lmo2, Mmp9, Traf1, Nupr1, Mllt3, Nfkbiz, Hist2h3c2*
	mmu05170	Human immunodeficiency virus 1 infection	9	4.41	*Bad, Gnb5, Gng5, Gngt2, Itpr2, Tap2, Tlr2, Mapk3, Tmem173*

**Table 3:** Protein modeling.

Proteins	Species	Protein Length (aa)	Model Templates (Query Cover, Identify)
LRP8	Mus musculus	498	5B4X_B (66%, 93.05%) 1IJQ_A (62%, 61.09%) 3V64_C (62%, 38.26%)
LDLR	Mus musculus	862	1IJQ_A (36%, 85.80%) 3V64_C (44%, 39.08%) 3SOQ_A (36%, 36.16%)
FCGR1	Mus musculus	404	4ZNE_A (65%, 73.31%) 4W4O_C (67%, 71.43%) 3RJD_A (64%, 73.66%)
HCAR2	Mus musculus	360	5XJM_A (31%, 38.94%) 5NJ6_A (64%, 34.44%) 5NDD_A (65%, 34.44%)
MMP9	Mus musculus	730	1L6J_A (58%, 82.16%) 1CK7_A (92%, 45.26%) 1EAK_A (57%, 58.47%)
EID3	Mus musculus	375	de novo
INSIG1	Mus musculus	259	de novo
YPEL3	Mus musculus	119	de novo

**Table 4:** Ramachandran plot analysis.

Proteins	Number of Residues in Favored Region	Number of Residues in Allowed Region	Number of Residues in Disallowed Region
LRP8	354 (80.3%)	82 (18.6%)	5 (1.1%)
LDLR	466 (81.3%)	97 (16.9%)	10 (1.7%)
FCGR1	302 (86.5%)	42 (12.0%)	5 (1.4%)
HCAR2	271 (83.4%)	50 (15.3%)	4 (1.2%)
MMP9	531 (89.5%)	59 (10.0%)	3 (0.5%)
EID3	322 (93.6%)	22 (6.4%)	0 (0.0%)
INSIG1	201 (93.5%)	14 (6.5%)	0 (0.0%)
YPEL3	96 (89.7%)	11 (10.3%)	0 (0.0%)

For Ldlr and Mmp9, part of the N-terminus was removed for subsequent molecular dynamics simulation.

**Table 5:** dcEF-sensitive genes.

Name Ensembl ID	Species Gene Type	Location Length	Expression Changes (dcEF vs. Control)	Protein Families	Function	Refs
*Fcgr1* (Fc receptor, IgG, high affinity I) (ENSMUSG00000015947.10)	Mus musculus Protein coding	Chr3 (2589 bp)	Up-regulated	PTHR11481 (14 genes)	Anticipated in the signal transduction of pain such as arthritis and joint inflammation.	[20,21]
*Ypel3* (yippee like 3) (ENSMUSG00000042675.15)	Mus musculus Protein coding	Chr7 (1057 bp)	Up-regulated	PTHR13847_SF179 (1 gene)	A novel tumor suppressor.	[22,23]
*Hcar2* (hydroxycarboxylic acid receptor 2) (ENSMUSG00000045502.6)	Mus musculus Protein coding	Chr5 (1930 bp)	Up-regulated	PTHR24231 (14 genes)	A novel negative regulator of macrophage activation.	[24,25,26]
*Mmp9* (matrix metallopeptidase 9) (ENSMUSG00000017737.2)	Mus musculus Protein coding	Chr2 (3175 bp)	Up-regulated	PTHR10201_SF30 (1 gene)	Involved in a wide range of biological functions including macrophage differentiation, inflammation, bone metabolism, and tumor invasion.	[27,28,29]
*Eid3* (EP300-interacting inhibitor of differentiation 3) (ENSMUSG00000109864.1)	Mus musculus Protein coding	Chr10 (1305 bp)	Down-regulated	PTHR16140_SF1 (1 gene)	A potent suppressor of nuclear receptor transcriptional activity.	[30]
*Lrp8* (low density lipoprotein receptor-related protein 8) (ENSMUSG00000028613.15)	Mus musculus Protein coding	Chr3 (3291 bp)	Down-regulated	PTHR10529_SF99 (1 gene)	A modulator of cell development and migration.	[31,32,33]
*Ldlr* (low density lipoprotein receptor) (ENSMUSG00000032193.9)	Mus musculus Protein coding	Chr9 (4549 bp)	Down-regulated	PTHR10529_SF195 (1 gene)	Deeply regulates the metabolism of lipids.	[34]
*Insig1* (insulin induced gene 1) (ENSMUSG00000045294.11)	Mus musculus Protein coding	Chr5 (2667 bp)	Down-regulated	PTHR15301 (2 genes)	Modulates innate immunity and cholesterol metabolism.	[35,36]

**Table 6:** GO analysis of dcEF-sensitive genes.

Genes	GO Analysis [37,38]
*Fcgr1*	MF: signaling receptor activity; signaling receptor binding.
	BP: cellular component organization; establishment of localization; immune system process; protein metabolic process; response to stimulus; signaling.
	CC: plasma membrane.
*Ypel3*	MF: none.
	BP: cell death; response to stimulus.
	CC: nucleus.
*Hcar2*	MF: carbohydrate derivative binding; signaling receptor activity.
	BP: cell death; establishment of localization; homeostatic process; immune system process; lipid metabolic process; response to stimulus; signaling.
	CC: plasma membrane.
*Mmp9*	MF: hydrolase.
	BP: cell death; cell differentiation; cell population proliferation; cellular component organization; establishment of localization; immune system process; protein metabolic process; response to stimulus; signaling; system development.
	CC: extracellular region.
*Eid3*	MF: none.
	BP: response to stimulus.
	CC: non-membrane-bounded organelle; nucleus; organelle lumen.
*Lrp8*	MF: cytoskeletal protein binding; signaling receptor activity.
	BP: cell death; cell differentiation; cellular component organization; establishment of localization; immune system process; nucleic acid-templated transcription; protein metabolic process; response to stimulus; signaling; system development.
	CC: cell projection; cytoskeleton; extracellular region; non-membrane-bounded organelle; plasma membrane; synapse.
*Ldlr*	MF: none.
	BP: cell differentiation; cellular component organization; establishment of localization; homeostatic process; immune system process; lipid metabolic process; protein metabolic process; response to stimulus; system development.
	CC: cytoplasmic vesicle; endosome; extracellular region; Golgi apparatus; plasma membrane; vacuole.
*Insig1*	MF: none.
	BP: cell differentiation; cellular component organization; establishment of localization; homeostatic process; lipid metabolic process; nucleic acid-templated transcription; response to stimulus; signaling; system development.
	CC: endoplasmic reticulum.

This information was from the Mouse Genome Database (MGD) at the Mouse Genome Informatics website, the Jackson Laboratory, Bar Harbor, Maine (URL: http://www.informatics.jax.org) [37,38] [date of retrieving data: August 12, 2019].

**Table 7:** dcEF-sensitive lncRNAs.

Name Ensembl ID	Species Gene Type	Location Length	Changes (dcEF vs. Control)	Function	Refs
*AI480526* (ENSMUSG00000090086.7)	Mus musculus LncRNA	Chr12 (1697 bp)	Down-regulated	May be related to the maturation of red blood cells. No specific function reported yet.	[39,40,41]
*Gm26520* (ENSMUSG00000097429.1)	Mus musculus LncRNA	Chr12 (1423 bp)	Down-regulated	No specific function reported yet.	[39]
*Gm26532* (ENSMUSG00000097296.1)	Mus musculus LncRNA	Chr8 (690 bp)	Down-regulated	No specific function reported yet.	[42]
*Gm28187* (ENSMUSG00000099375.1)	Mus musculus LncRNA	Chr1 (2134 bp)	Down-regulated	May be related to mammary tumor cell proliferation and migration.	[43]
*Snhg20* (ENSMUSG00000086859.4)	Mus musculus LncRNA	Chr11 (627 bp)	Down-regulated	Promote tumor cell proliferation.	[39,44,45]

**Table 8:** Comparison of EF-induced gene expression pathway changes in human and mouse samples.

Pathway Name	Fold Change or *p*-Value					
	HDF-a dcEF: 100 mV/mm 1 h [47]	Human Adult Epidermal Keratinocytes dcEF: 100 mV/mm 1 h [48]	CL1–5 dcEF: 300 mV/mm 2 h [49]	U87 mg dcEF: 250 mV/mm 8 h [50]	DAOY dcEF: 250 mV/mm 8 h [50]	RAW 264.7 dcEF: 200 mV/mm 4 h
Transcription	YAF2: 2.6 JMJD1C: 2.4 ZBTB24: 2.0 ZNF15L1: 1.8 LRRFIP1: 1.8 ZNF207: 1.7 RNF12: 1.7 SIX1: 1.5 FOXJ1: 1.4 ITPR1: 1.6 IL1RAPL1: 1.4 MAPK1: 1.4	WNK1: 1.8 RAB6IP2: 1.7 PICALM: 1.7 ATP11B: 1.7 CENTG2: 1.6 SEC15L2: 1.6 AP3B1: 1.5 ATP6V0E: 1.5 FLVCR: 1.5	NFYA: 1.16 NFYC: 1.27	Gene: NA *p* = 0.00000718	Gene: NA *p* = 1.23 × 10^−8^	Gadd45a: 3.25 Ddit3: 3.48 Fcgr1: 3.18 Lmo2: 1.72 Mmp9: 4.64 Traf1: 2.95 Nupr1: 3.46 Mllt3: 1.83 Nfkbiz: 2.63 Hist2h3c2: 3.51

HDF-a: the human adult dermal fibroblast cell line; CL1–5: the human lung cancer cell line; U87 mg: the human glioblastoma cell line; DAOY: the human medulloblastoma cell line; RAW 264.7: the murine macrophage cell line; and NA: not available. Gene name list: NFYA: nuclear transcription factor Y, alpha; NFYC: nuclear transcription factor Y, gamma; YAF2: YY1 associated factor 2; JMJD1C: Jumonji domain containing 1C; ZBTB24: zinc finger and BTB domain (Broad-Complex, Tramtrack and Bric a brac) containing 24; ZNF15L1: zinc finger protein 708 (KOX8); LRRFIP1: leucine rich repeat (in FLII) interacting protein 1; ZNF207: zinc finger protein 207; RNF12: ring finger protein 12; SIX1: sine oculis homeobox homolog 1 (*Drosophila*); FOXJ1: Forkhead box J1; ITPR1: inositol 1,4,5-triphosphate receptor, type 1; IL1RAPL1: interleukin 1 receptor accessory protein-like 1; MAPK1: mitogen-activated protein kinase 1; WNK1: WNK lysine deficient protein kinase 1; RAB6IP2: RAB6 interacting protein 2; PICALM: phosphatidylinositol binding clathrin assembly protein; ATP11B: ATPase, Class VI, type 11B; CENTG2: centaurin, gamma 2; SEC15L2: SEC15-like 2 (S. cerevisiae); AP3B1: adaptor-related protein complex 3, beta 1 subunit; ATP6V0E: ATPase, H^+^ transporting, lysosomal 9 kDa, V0 subunit e; FLVCR: feline leukemia virus subgroup C cellular receptor; Gadd45a: growth arrest and DNA-damage-inducible 45 alpha; Ddit3: DNA-damage inducible transcript 3; Fcgr1: Fc receptor, IgG, high affinity I; Lmo2: LIM domain only 2; Mmp9: matrix metallopeptidase 9; Traf1: TNF receptor-associated factor 1; Nupr1: nuclear protein transcription regulator 1; Mllt3: myeloid/lymphoid or mixed-lineage leukemia; translocated to, 3; Nfkbiz: nuclear factor of kappa light polypeptide gene enhancer in B cells inhibitor, zeta; Hist2h3c2: histone cluster 2, H3c2.

**Table 9:** Primers used for qPCR.

Primers		Sequences (5′ to 3′)	Product (bp)
*GAPDH*	Forward	TGTGTCCGTCGTGGATCTGA	150
	Reverse	TTGCTGTTGAAGTCGCAGGAG	
*Fcgr1*	Forward	GGTTCCTCAATGCCAAGT	128
	Reverse	TTATTCTTCCATCCGTGACA	
*Ypel3*	Forward	ACCTCTTCAACTCTGTAGTG	154
	Reverse	TACTTCTGGCTGCTCTCA	
*Hcar2*	Forward	ACTGTCCACCTCCTCTATAC	194
	Reverse	TGTCTGTCCATCTGTCTCT	
*Mmp9*	Forward	AGATTCTCCGTGTCCTGTA	163
	Reverse	AGTCTGACCTGAACCATAAC	
*Eid3*	Forward	TAGCGACTGCGATGATAG	187
	Reverse	CATATCTCCACTTCCTTCCA	
*Lrp8*	Forward	GACAAGGAGTAAGAAGAATGAG	115
	Reverse	ACAGCAGCGAGTGAATAC	
*Ldlr*	Forward	GTGTGAAGATATTGACGAGTG	162
	Reverse	TTGGTGAAGAGCAGATAGC	
*Insig1*	Forward	CTAAGAGTGAGTCGCTGTC	196
	Reverse	GTGTTGTGTTCTATGCTGTC	
*AI480526*	Forward	GGTCTGTGCTTAGTCTCTG	156
	Reverse	TGCTACCTGGAACCTTGT	
*Gm26520*	Forward	CAATGGTGGAGGAGAGGA	124
	Reverse	GATGCTAGTGAGGTCAAGAA	
*Gm26532*	Forward	GCCTCAGGATATGAACAGAT	131
	Reverse	TCAGCCAGTTCCAATTAGTC	
*Gm28187*	Forward	CCAACACATATACAGCAGAAG	124
	Reverse	TCCACCTCCTATCCTCCT	
*Snhg20*	Forward	CTGGCTGCTTCTGTGTTG	123
	Reverse	TGCTTCCGTCTAGTCGTT	

## Supplementary Materials

The following are available online at https://www.mdpi.com/1422-0067/21/12/4505/s1, Figure S1: The enrichment analysis was performed by Metascape. (A) Network of enriched terms (colored by cluster ID). (B) Network of enriched terms (colored by *p*-value), Figure S2: Functional enrichment analysis by KEGG. (A) Detailed information of sub-pathway (steroid biosynthesis) in the KEGG. (B) KEGG pathways with the most significant enrichment result. The abscissa was the ratio of the number of differentially expressed genes, which annotated the KEGG pathway to the total number of differentially expressed genes, Figure S3: The 3 sub-networks from the protein–protein interaction network. (A) Module 1. (B) The enriched pathways of module 1. (C) Module 2. (D) The enriched pathways of module 2. (E) Module 3. (F) The enriched pathways of module 3, Figure S4: Superposition of the primarily modeled structure (gray) and the MD-optimized protein structure (violet), Figure S5: RMSF comparison plots of DEG proteins during molecular dynamics simulation (at least > 100 ns). The RMSF of *Eid3, Lrp8, Ldlr, Insig1, Fcgr1, Ypel3, Hcar2,* and *Mmp9* are shown. For each protein, 0v (black), 0.01v (red), 0.05v (green), and 0.5v (blue) are presented on one map, Figure S6: Gyration radius comparison plots of DEG proteins during molecular dynamics simulation (at least > 100 ns). The Gyration radius of *Eid3, Lrp8, Ldlr, Insig1, Fcgr1, Ypel3, Hcar2,* and *Mmp9* are shown. For each protein, 0v (black), 0.01v (red), 0.05v (green), and 0.5v (blue) are presented on one map, Table S1: RMSD value average analysis, Table S2: RMSF value average analysis, Table S3: R*g* value average analysis, Table S4: RMSD value of protein model and MD-optimized protein model.

## Abbreviations

BP	biological process
cBioPortal	cBio Cancer Genomics Portal
CC	cell component
DEGs	differentially expressed genes
FC	fold control
GO	Gene Ontology
KEGG	Kyoto Encyclopedia of Genes and Genomes pathway
MCODE	Molecular Complex Detection
MF	molecular function
PPI	protein–protein interaction
RMSD	root mean square deviation
RMSF	root mean square fluctuation
DMEM	Dulbecco’s Modified Eagle’s medium
FBS	fetal bovine serum
DOPE	discrete optimized protein energy
dcEF	direct current electric field
