# Supplementary material for: Hub Proteins Involved in RAW 264.7 Macrophages Exposed to Direct Current Electric Field
Source: Int J Mol Sci. 2020 Jun 24;21(12):4505. doi: 10.3390/ijms21124505 (PMC7352442; doi:10.3390/ijms21124505)
Supplement: Supplementary file 1 [file ijms-21-04505-s001.zip › Supplementary/Supplementary file 1-Figures.docx]

**Supplementary Information**

Hub proteins involved in RAW 264.7 macrophages exposed to direct current electric field

Huijuan Li ^1^, Shibin Liu ^1*^, Yongqian Du ^1^, Jie Tan ^1^, Jiezhang Luo ^1^, and Yulong Sun ^2*^

Affiliations:

^1^ School of Electronics and Information, Northwestern Polytechnical University, Xi’an, 710072, China

^2^ Key Laboratory for Space Biosciences & Biotechnology, School of Life Sciences, Northwestern Polytechnical University, Xi’an, 710072, China

Emails:

*Corresponding authors:

Shibin Liu, Ph.D.

Address: School of Electronics and Information, Northwestern Polytechnical University, 127 Youyi Xilu, Xi’ an Shaan Xi Province, 710072, PR China

Tel.: +86 29 88491653, E-mail: liushibin@nwpu.edu.cn

Yulong Sun, Ph.D.

Address: School of Life Sciences, Northwestern Polytechnical University, 127 Youyi Xilu, Xi’ an Shaan Xi Province, 710072, PR China

Tel.: +86 29 88460332, E-mail: yulongsun@nwpu.edu.cn

**Figure of contents**

1. **Figures**

.

A

**
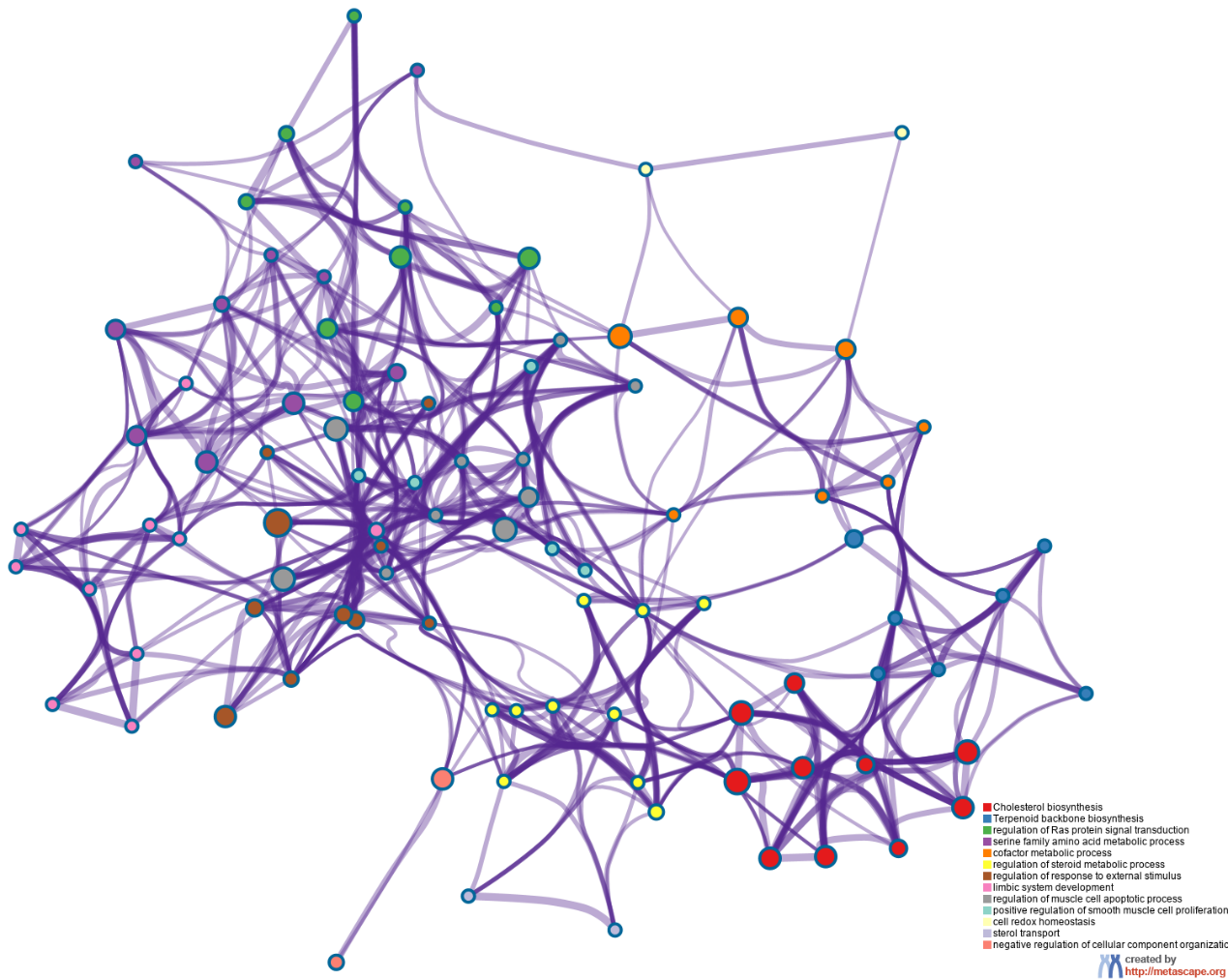
**

B


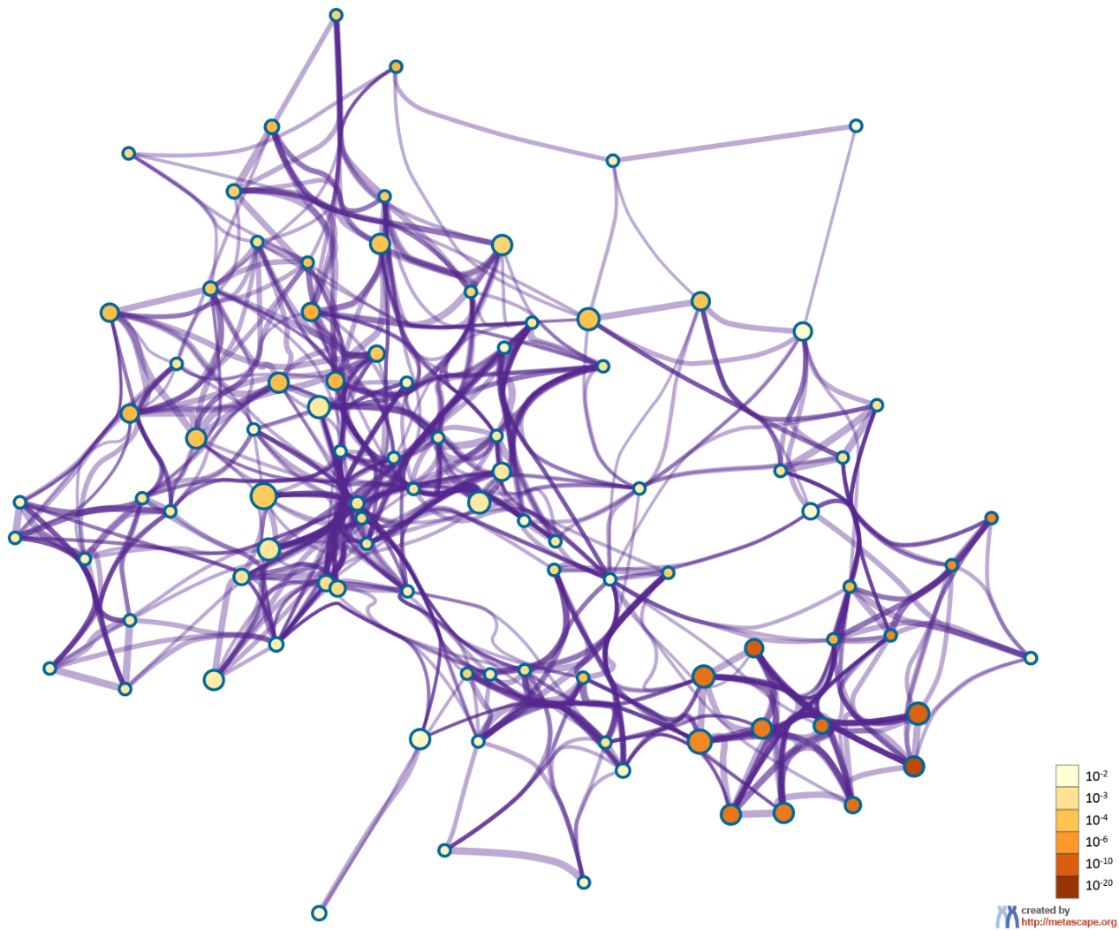


**Figure S1** The enrichment analysis was performed by Metascape. (A) Network of enriched terms (colored by cluster ID). (B) Network of enriched terms (colored by p-value).

A


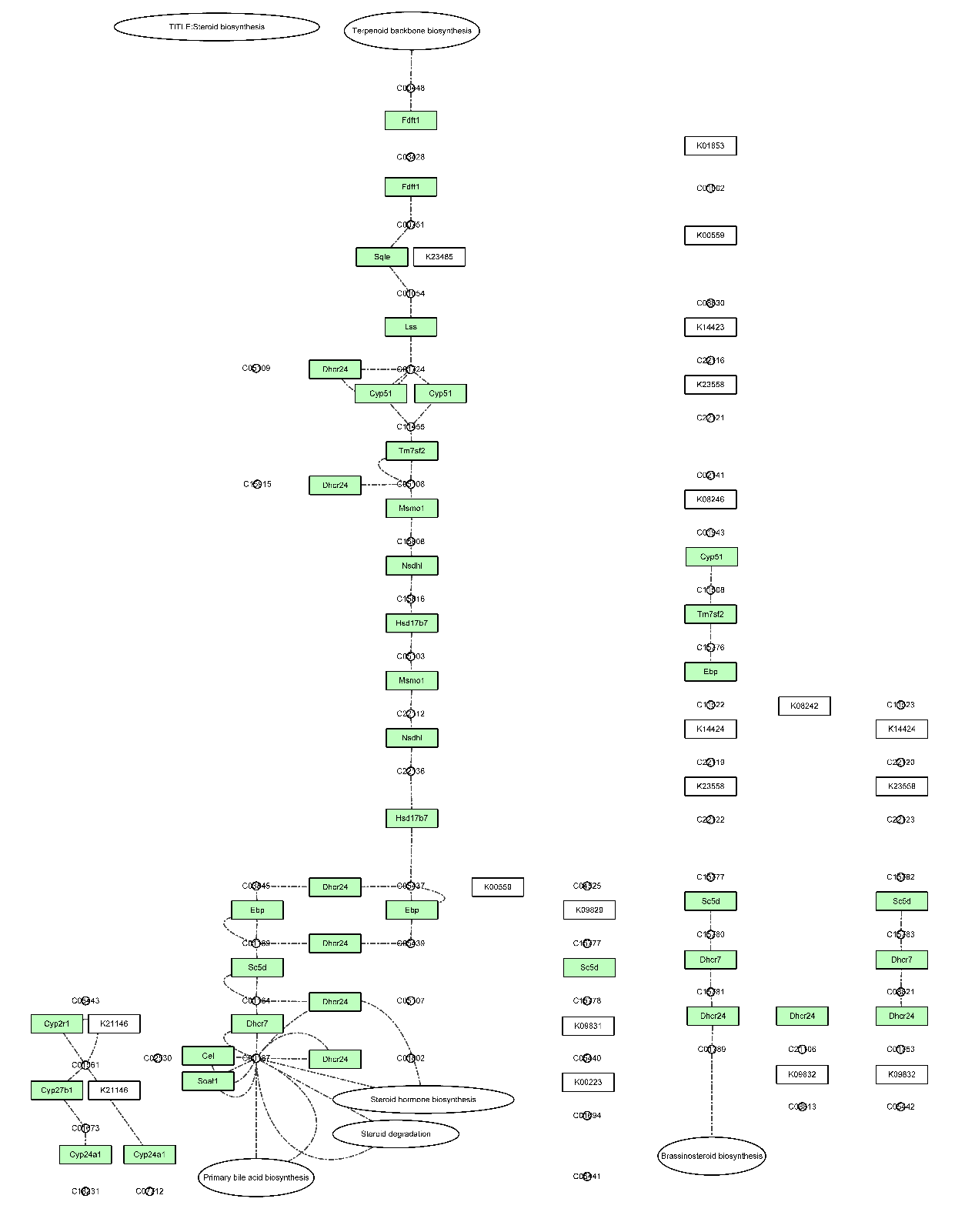


B


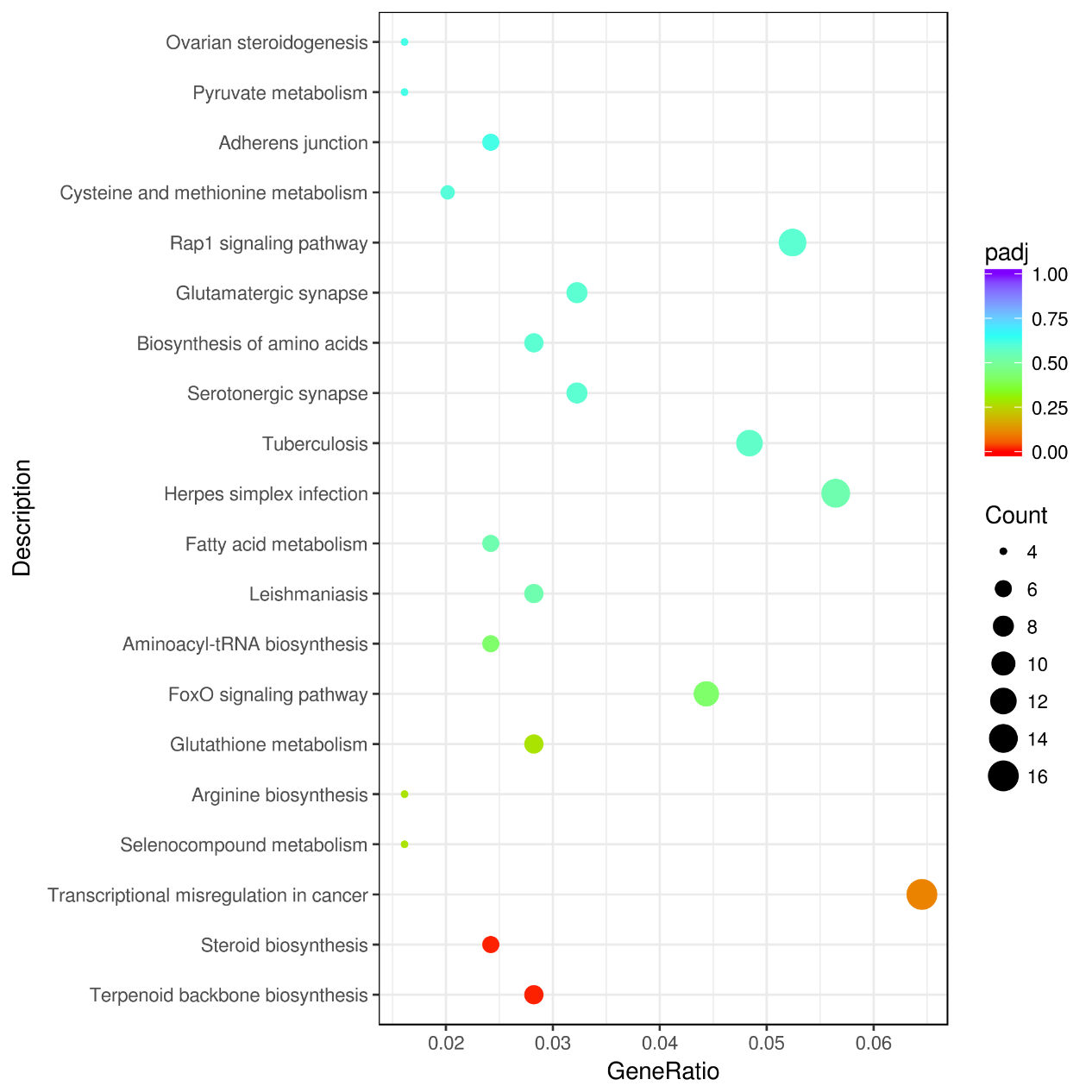


**Figure S2** Functional enrichment analysis by KEGG. (A) Detailed information of sub-pathway (steroid biosynthesis) in the KEGG. (B) KEGG pathways with the most significant enrichment result. The abscissa was the ratio of the number of differentially expressed genes, which annotated the KEGG pathway to the total number of differentially expressed genes.

A


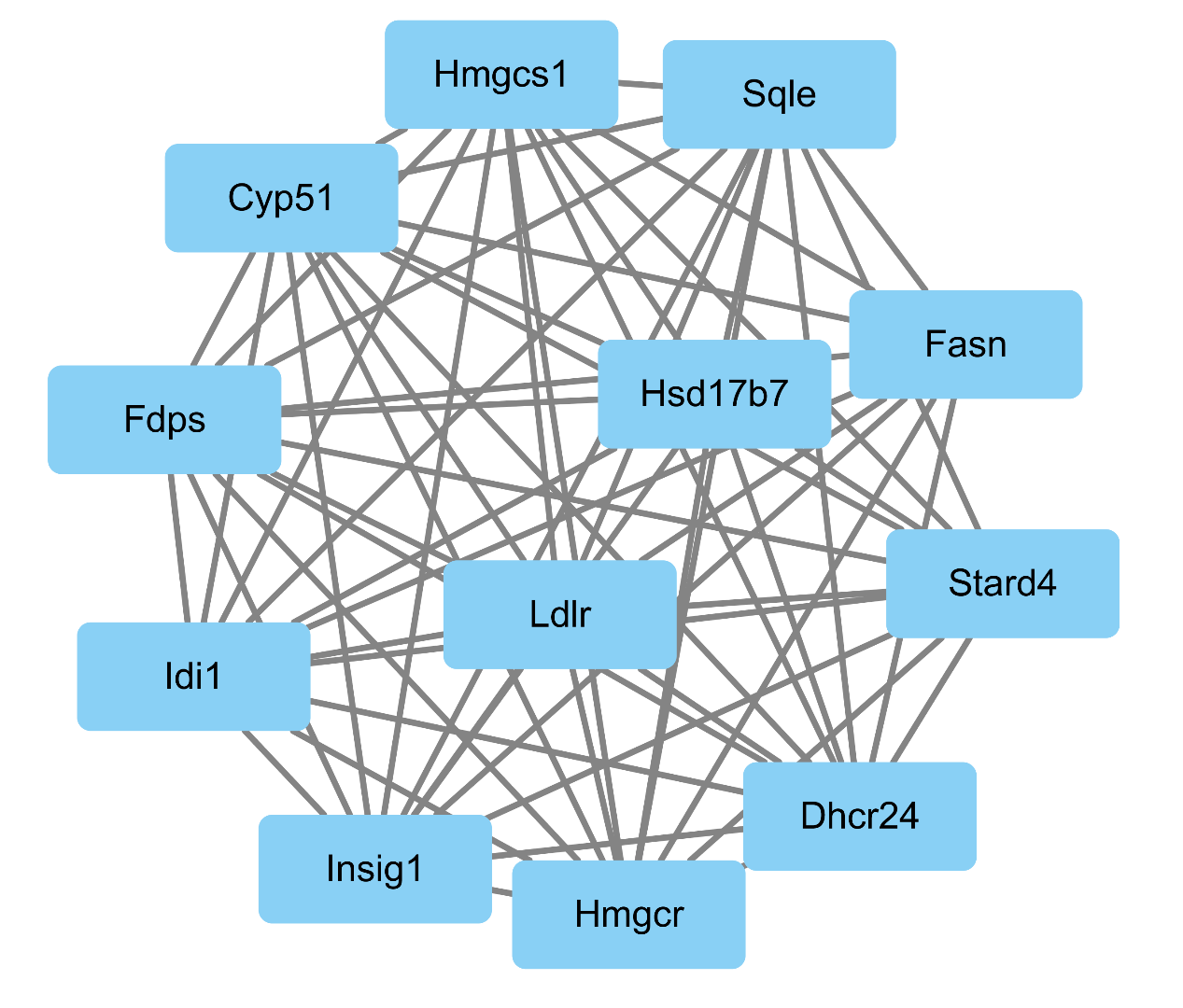


B

| GeneSet | P-value | Correct p-value | Nodes |
| --- | --- | --- | --- |
| cholesterol metabolic process | 3.09E-25 | 8.12E-23 | *Fdps Idi1 Sqle Hmgcs1 Insig1 Dhcr24 Hmgcr Hsd17b7 Ldlr Cyp51* |
| sterol metabolic process | 8.30E-25 | 1.09E-22 | *Fdps Idi1 Sqle Hmgcs1 Insig1 Dhcr24 Hmgcr Hsd17b7 Ldlr Cyp51* |
| cholesterol biosynthetic process | 2.96E-23 | 2.60E-21 | *Fdps Idi1 Hmgcs1 Insig1 Dhcr24 Hmgcr Hsd17b7 Cyp51* |

C


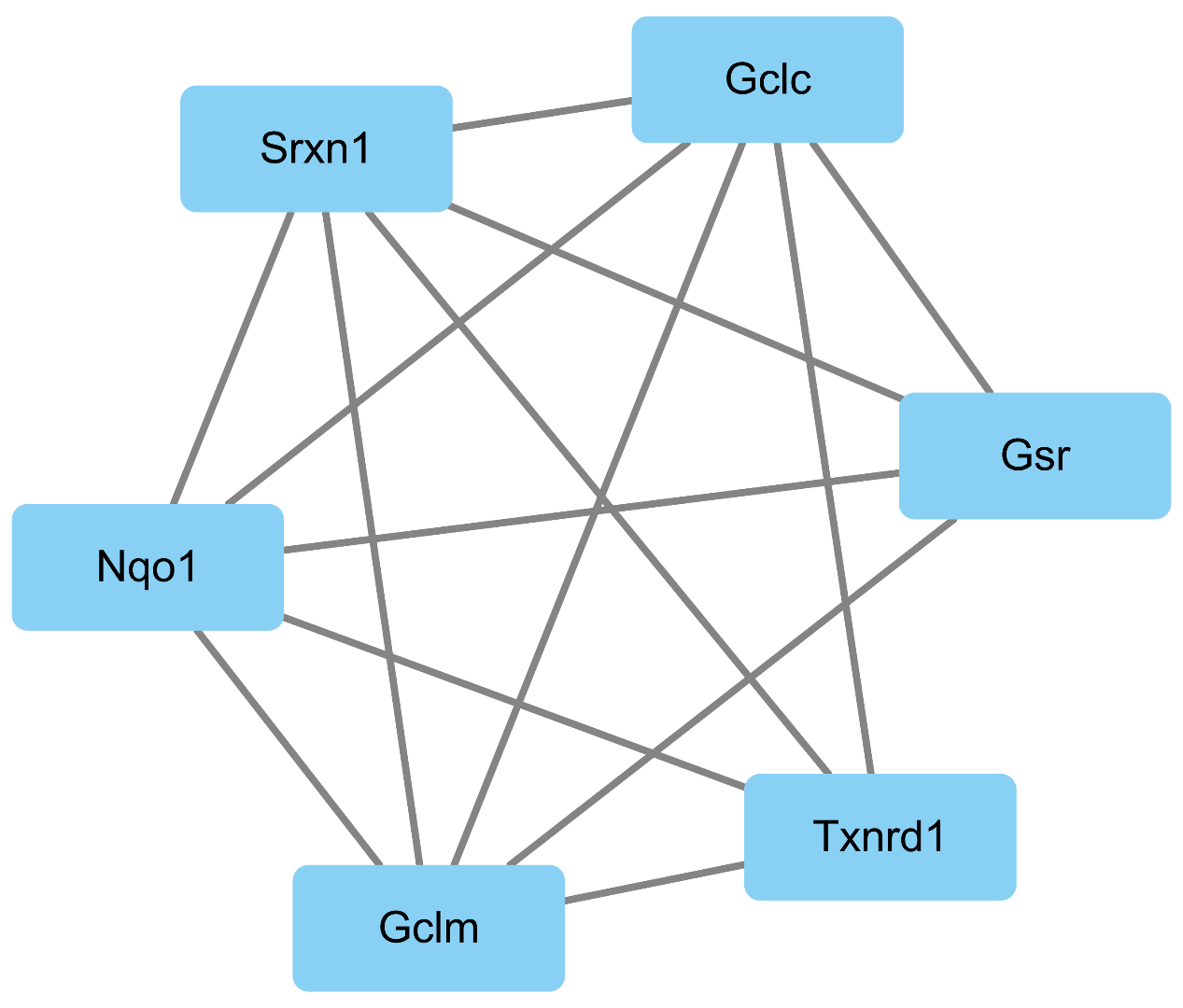


D

| GeneSet | P-value | Correct p-value | Nodes |
| --- | --- | --- | --- |
| response to oxidative stress | 3.93E-12 | 6.01E-10 | *Nqo1 Gclc Srxn1 Txnrd1 Gclm* |
| glutathione metabolic process | 2.00E-08 | 1.53E-06 | *Gclc Gsr Gclm* |
| oxidation reduction | 3.06E-08 | 1.56E-06 | *Nqo1 Srxn1 Txnrd1 Gsr Gclm* |

E


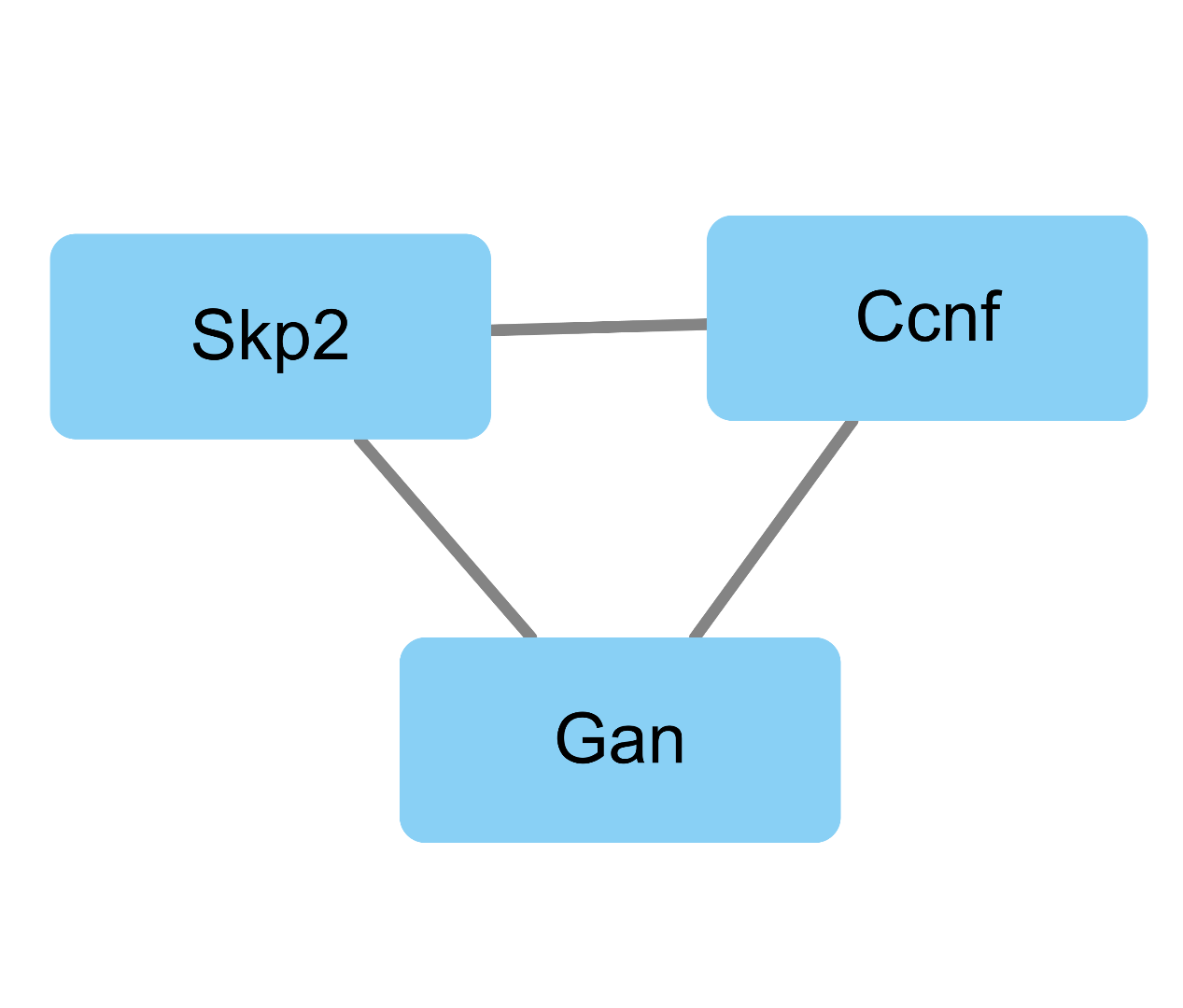


F

| GeneSet | P-value | Correct p-value | Nodes |
| --- | --- | --- | --- |
| mitotic cell cycle | 1.97E-04 | 6.28E-03 | *Ccnf Skp2* |
| negative regulation of survival gene product expression | 2.07E-04 | 6.28E-03 | *Skp2* |
| re-entry into mitotic cell cycle | 3.11E-04 | 6.28E-03 | *Ccnf* |

**Figure S3** The 3 sub-networks from the protein–protein interaction network. (A) Module 1. (B) The enriched pathways of module 1. (C) Module 2. (D) The enriched pathways of module 2. (E) Module 3. (F) The enriched pathways of module 3.


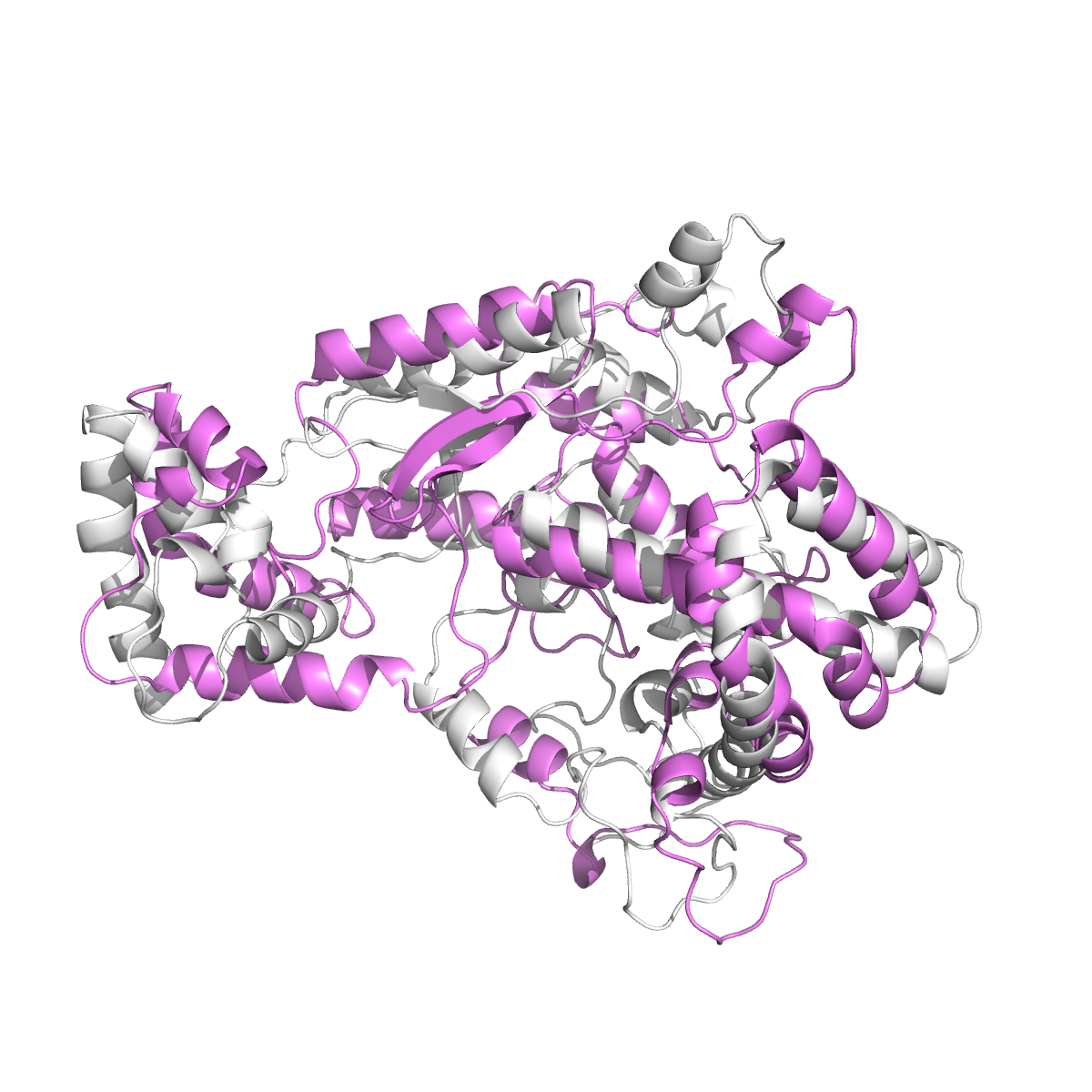


A

***Eid3***


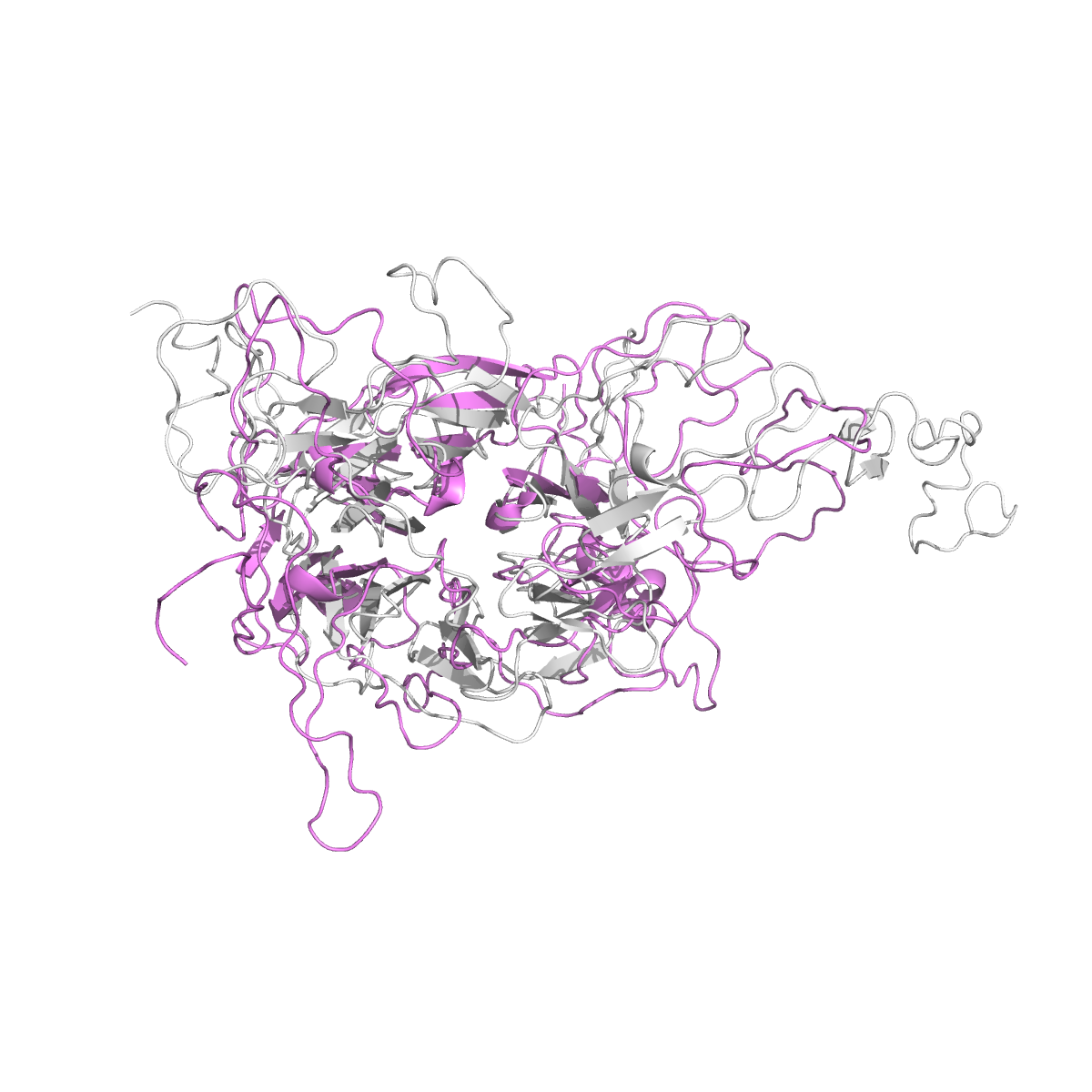


B

***Lrp8***


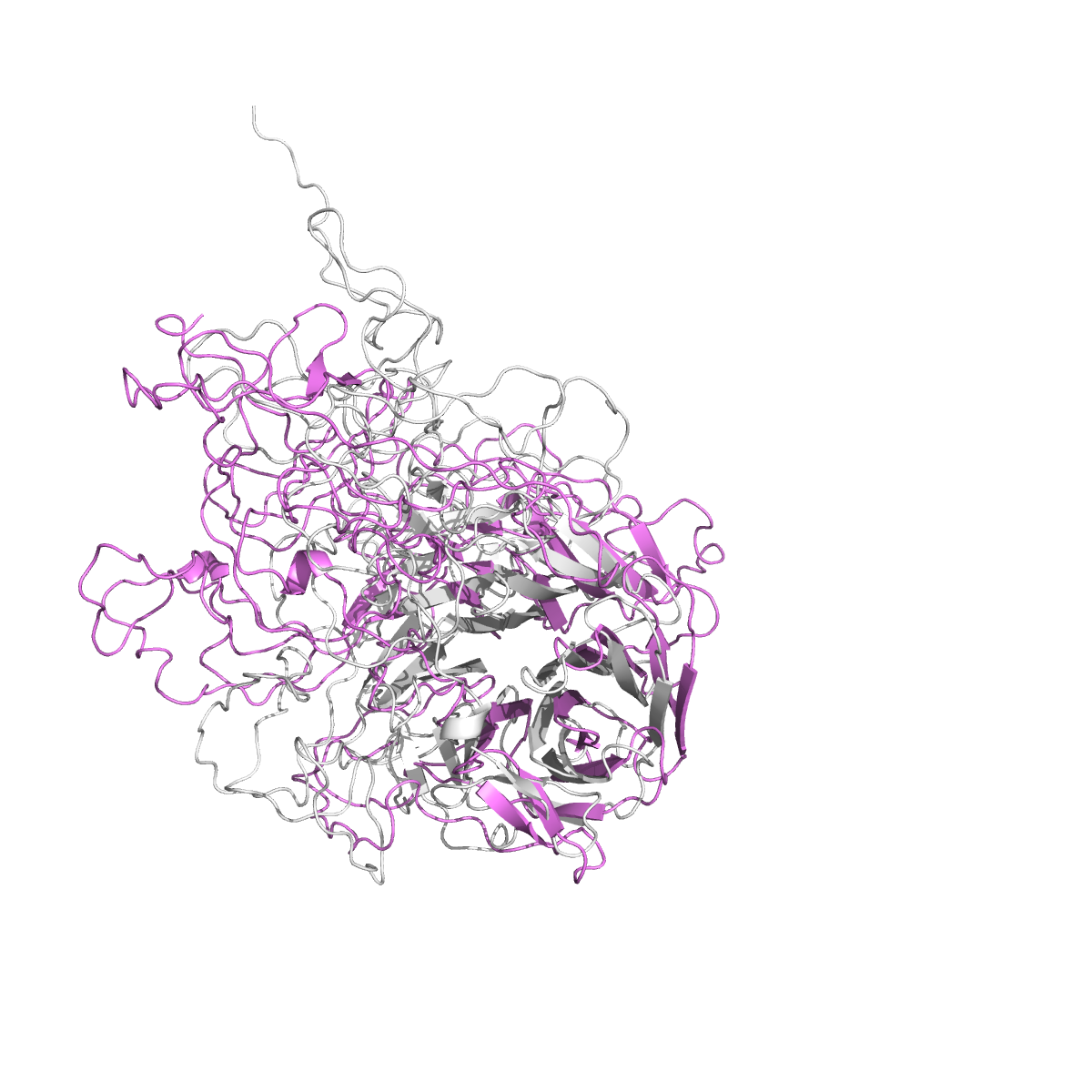


C

***Ldlr***


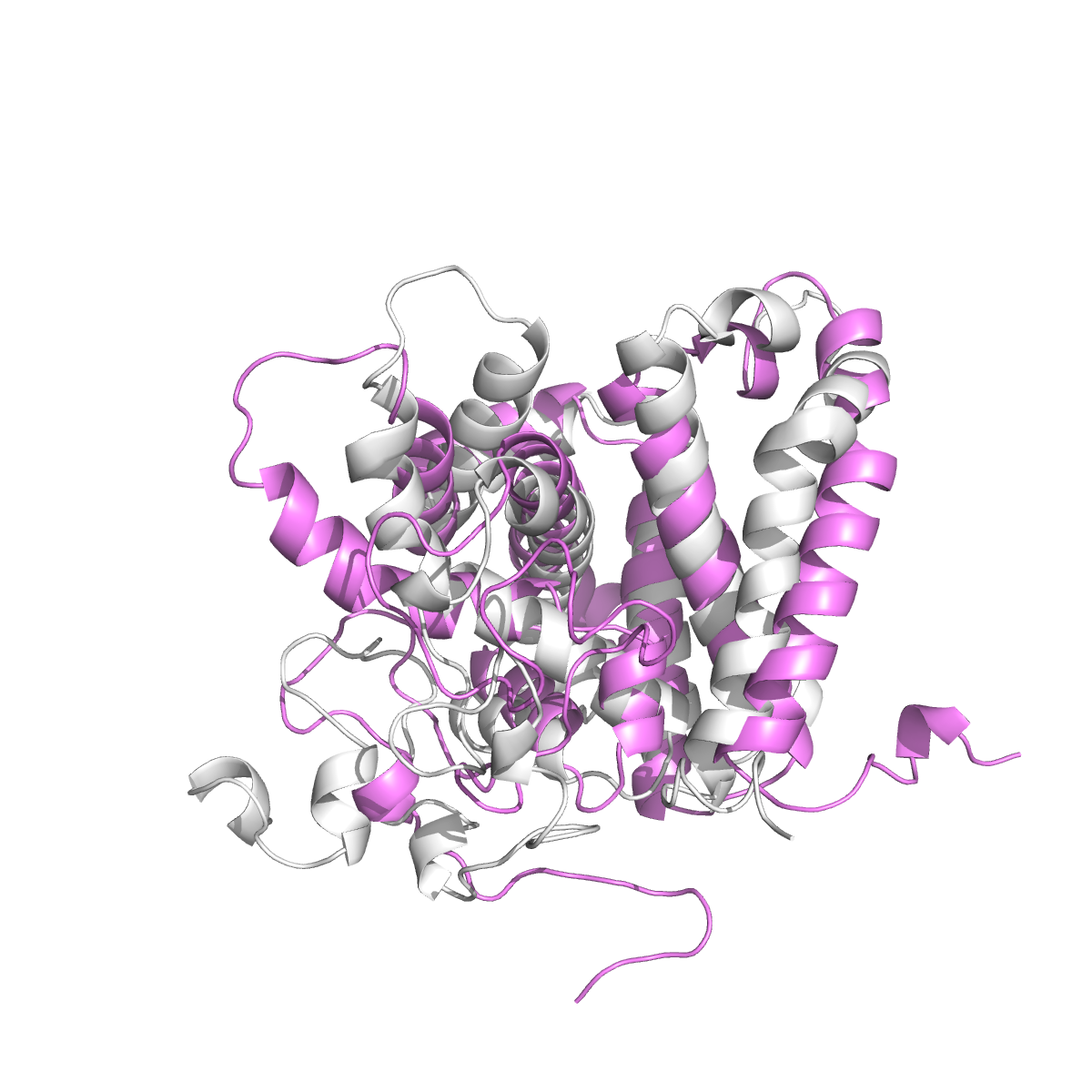


D

***Insig1***


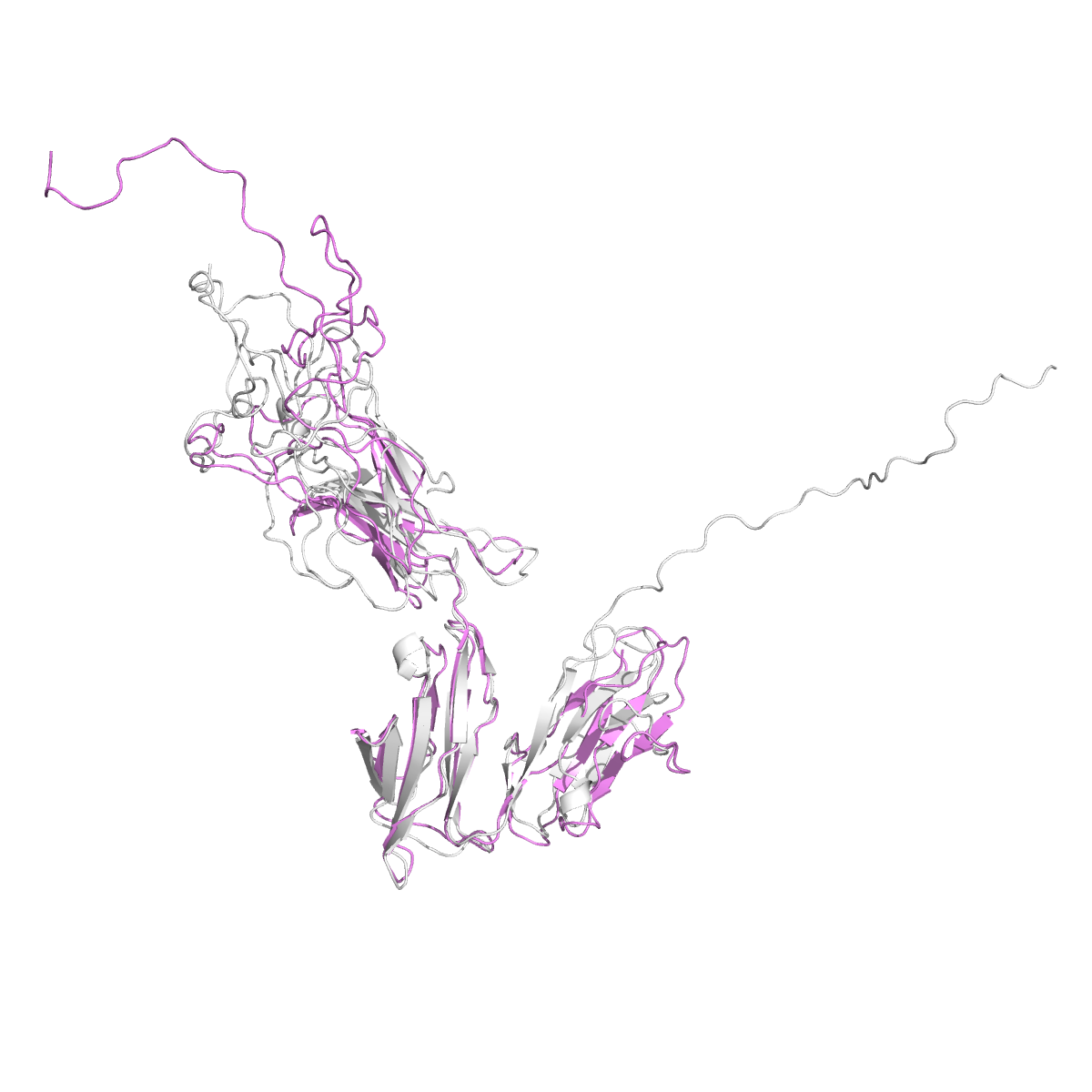


E

***Fcgr1***


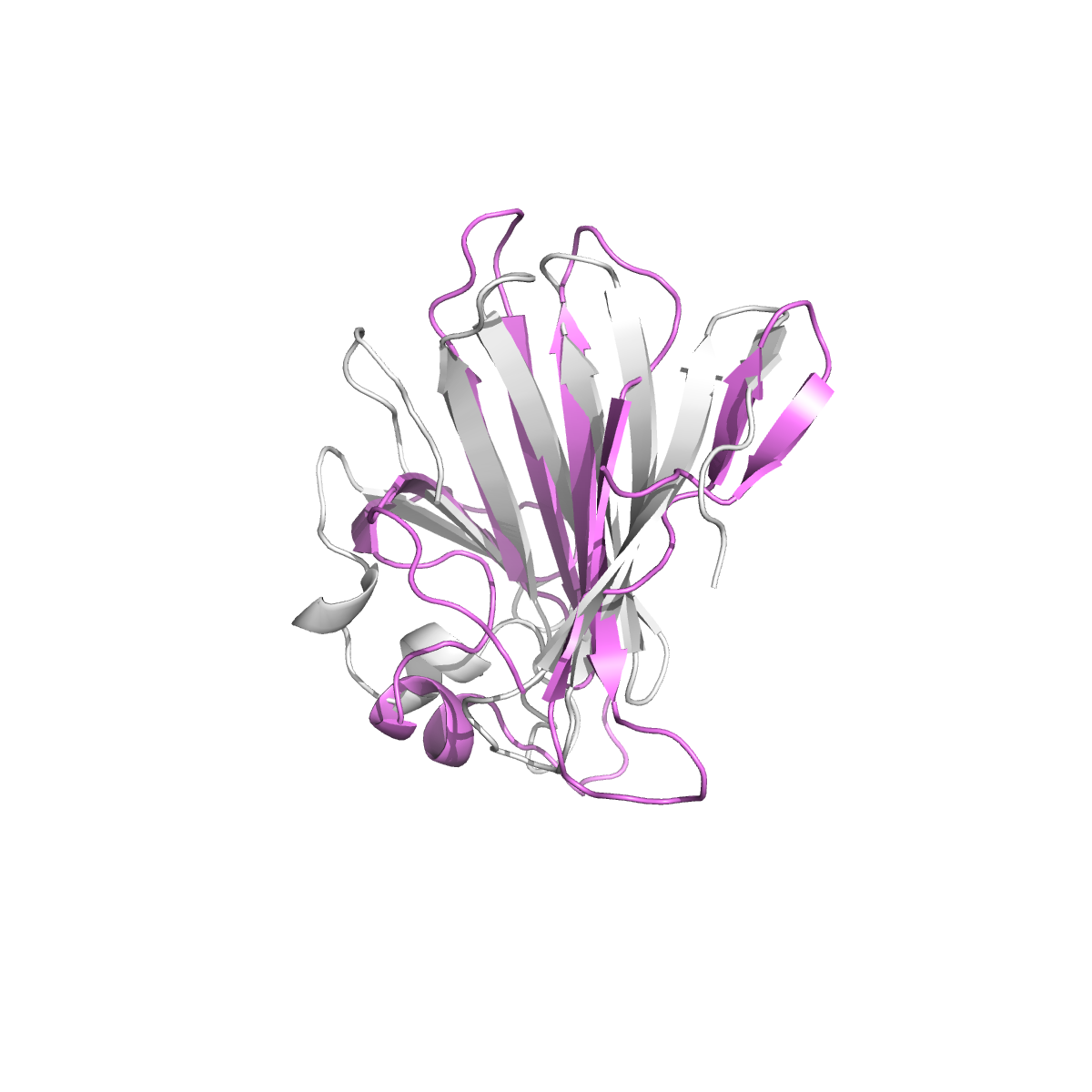


F

***Ypel3***


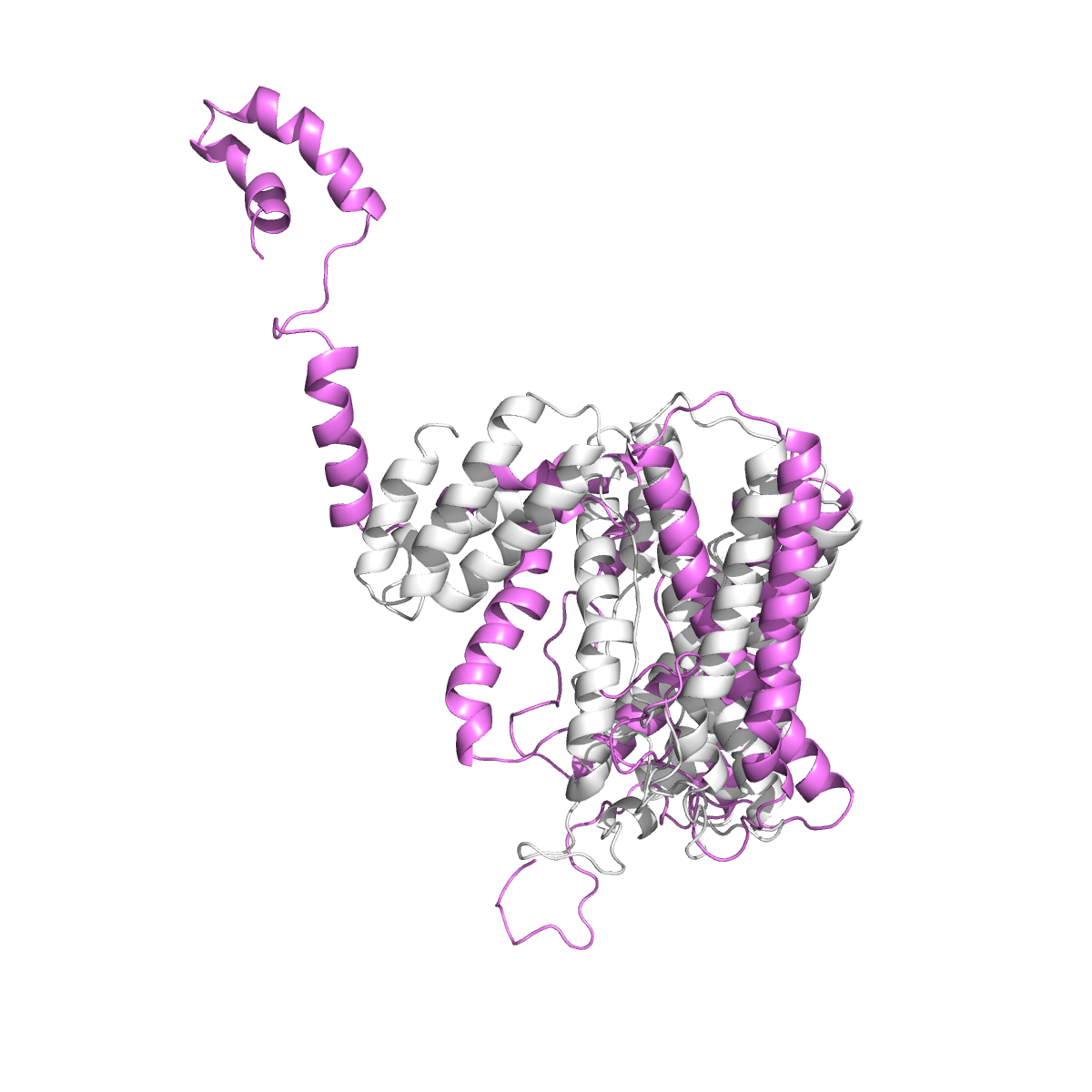


G

***Hcar2***


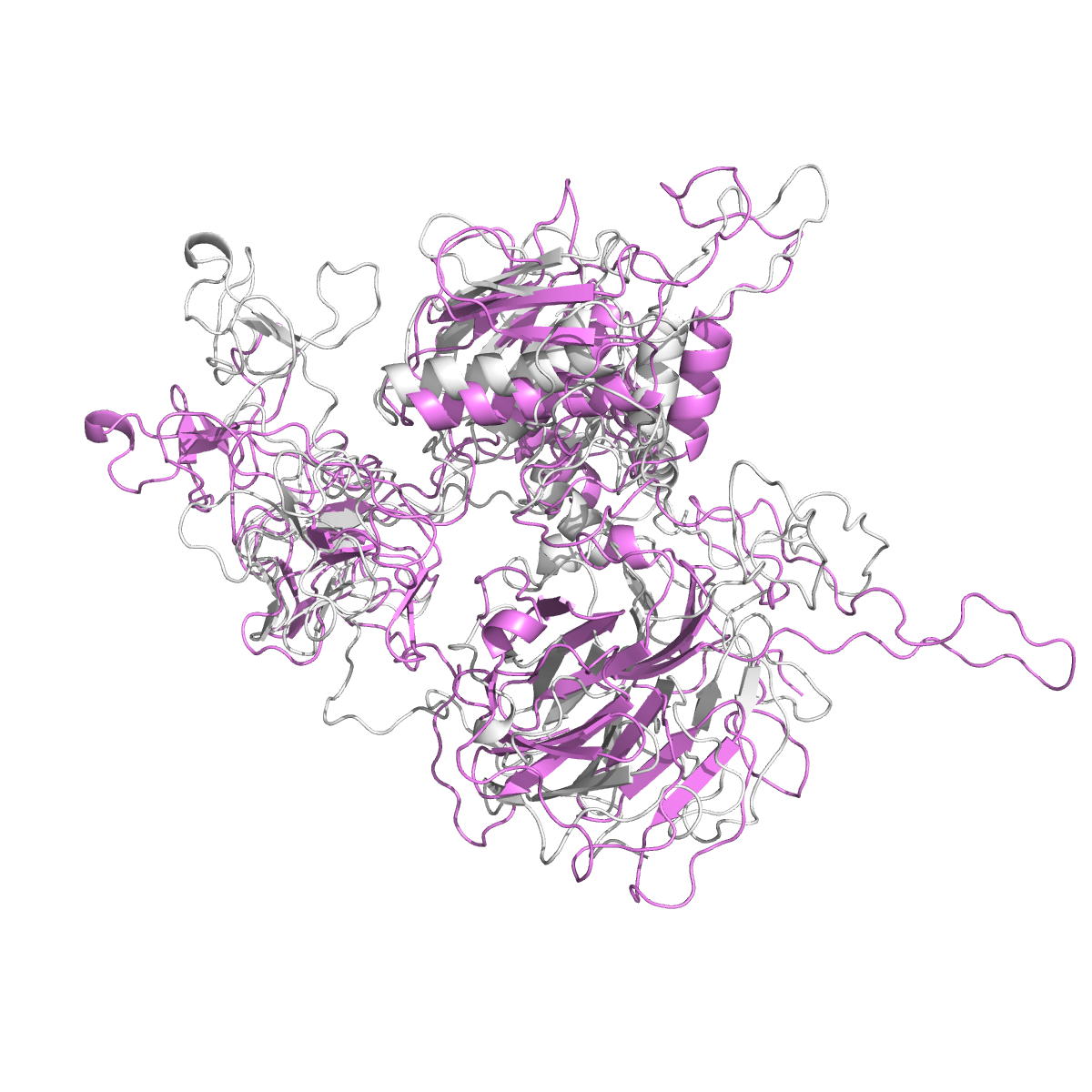


H

***Mmp9***

**Figure S4** Superposition of the primarily modeled structure (gray) and the MD-optimized protein structure (violet).


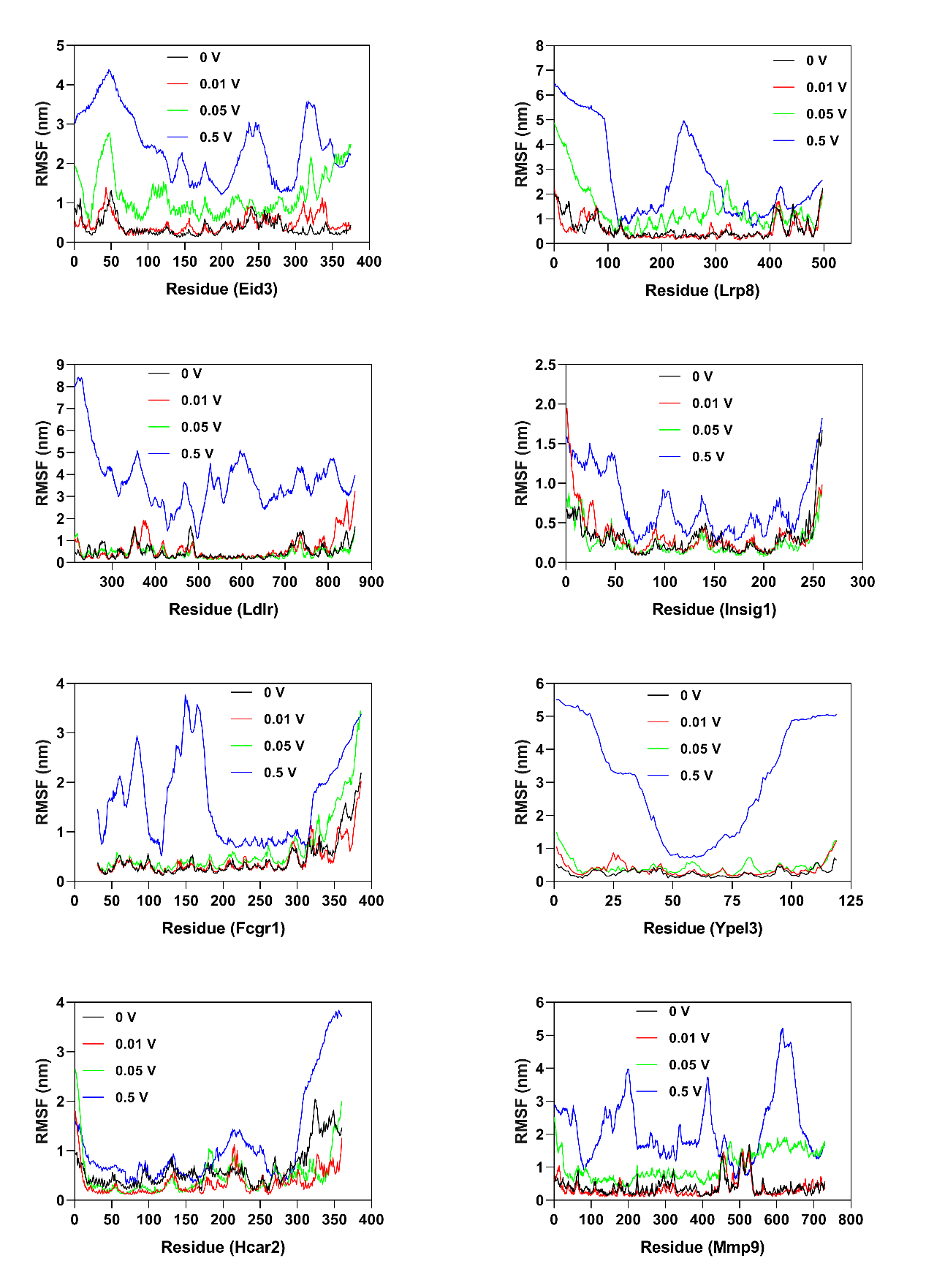


**Figure S5** RMSF comparison plots of DEGs protein during molecular dynamics simulation (at least > 100 ns). The RMSF of *Eid3, Lrp8, Ldlr, Insig1, Fcgr1, Ypel3, Hcar2,* and *Mmp9* are shown. For each protein, 0v (black), 0.01v (red), 0.05v (green), and 0.5v (blue) are presented on one map.


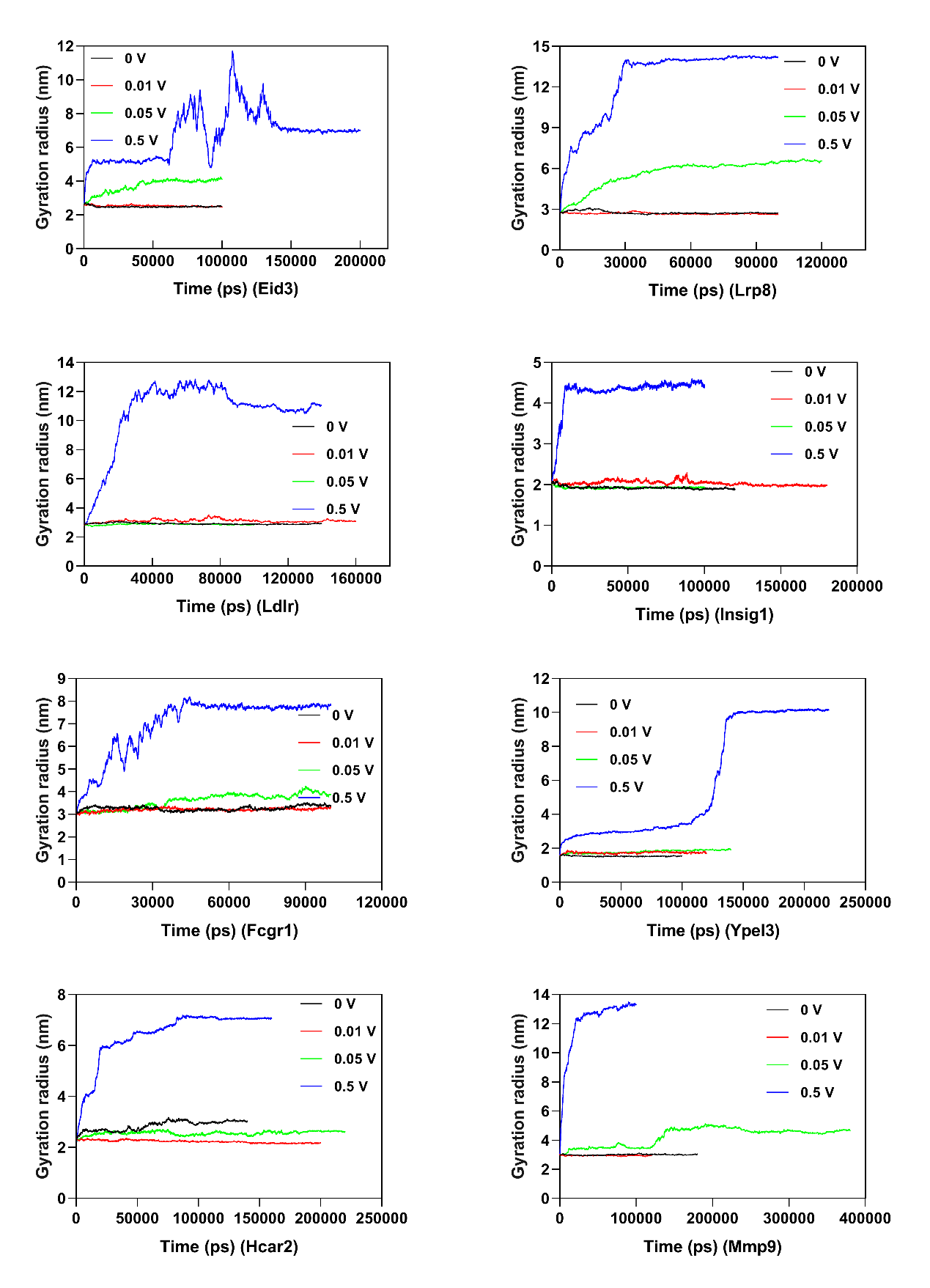


**Figure S6** Gyration radius comparison plots of DEGs protein during molecular dynamics simulation (at least > 100 ns). The Gyration radius of *Eid3, Lrp8, Ldlr, Insig1, Fcgr1, Ypel3, Hcar2,* and *Mmp9* are shown. For each protein, 0v (black), 0.01v (red), 0.05v (green), and 0.5v (blue) are presented on one map.
