# Supplementary material for: Hub Proteins Involved in RAW 264.7 Macrophages Exposed to Direct Current Electric Field
Source: Int J Mol Sci. 2020 Jun 24;21(12):4505. doi: 10.3390/ijms21124505 (PMC7352442; doi:10.3390/ijms21124505)
Supplement: Supplementary file 1 [file ijms-21-04505-s001.zip › Supplementary/Supplementary file 2-Tables.docx]

**Supplementary Information**

Hub proteins involved in RAW 264.7 macrophages exposed to direct current electric field

Huijuan Li ^1^, Shibin Liu ^1*^, Yongqian Du ^1^, Jie Tan ^1^, Jiezhang Luo ^1^, and Yulong Sun ^2*^

Affiliations:

^1^ School of Electronics and Information, Northwestern Polytechnical University, Xi’an, 710072, China

^2^ Key Laboratory for Space Biosciences & Biotechnology, School of Life Sciences, Northwestern Polytechnical University, Xi’an, 710072, China

Emails:

*Corresponding authors:

Shibin Liu, Ph.D.

Address: School of Electronics and Information, Northwestern Polytechnical University, 127 Youyi Xilu, Xi’ an Shaan Xi Province, 710072, PR China

Tel.: +86 29 88491653, E-mail: liushibin@nwpu.edu.cn

Yulong Sun, Ph.D.

Address: School of Life Sciences, Northwestern Polytechnical University, 127 Youyi Xilu, Xi’ an Shaan Xi Province, 710072, PR China

Tel.: +86 29 88460332, E-mail: yulongsun@nwpu.edu.cn

**Table of contents**

1. **Tables**

.

**Table S1** RMSD value average analysis

| Gene | Model | 0.01 v | 0.05 v | 0.5 v |
| --- | --- | --- | --- | --- |
| *Fcgr1* | 1.061 | 0.803 | 1.42 | 5.919 |
| *Hcar2* | 1.435 | 0.9028 | 1.185 | 5.509 |
| *Mmp9* | 0.8668 | 0.9583 | 2.665 | 10.59 |
| *Lrp8* | 1.579 | 1.331 | 3.897 | 10.6 |
| *Ldlr* | 1.419 | 1.392 | 1.256 | 9.508 |
| *Eid3* | 0.9512 | 1.129 | 2.609 | 5.711 |
| *Insig1* | 0.8677 | 0.7596 | 0.7701 | 3.531 |
| *Ypel3* | 0.5169 | 0.824 | 0.8899 | 5.164 |

**Table S2** RMSF value average analysis

| Gene | Model | 0.01 v | 0.05 v | 0.5 v |
| --- | --- | --- | --- | --- |
| *Fcgr1* | 0.4447 | 0.4164 | 0.6484 | 1.641 |
| *Hcar2* | 0.6277 | 0.3557 | 0.4869 | 1.106 |
| *Mmp9* | 0.405 | 0.3499 | 1.043 | 2.157 |
| *Lrp8* | 0.5995 | 0.5496 | 1.362 | 2.709 |
| *Ldlr* | 0.4813 | 0.6141 | 0.4312 | 3.739 |
| *Eid3* | 0.3829 | 0.4678 | 1.186 | 2.434 |
| *Insig1* | 0.3046 | 0.3436 | 0.2512 | 0.7306 |
| *Ypel3* | 0.2532 | 0.3665 | 0.4452 | 3.109 |

**Table S3** R*g* value average analysis

| Gene | Model | 0.01 v | 0.05 v | 0.5 v |
| --- | --- | --- | --- | --- |
| *Fcgr1* | 3.263 | 3.209 | 3.597 | 6.996 |
| *Hcar2* | 2.856 | 2.231 | 2.562 | 6.422 |
| *Mmp9* | 3.001 | 2.936 | 4.264 | 12.08 |
| *Lrp8* | 2.76 | 2.688 | 5.699 | 12.48 |
| *Ldlr* | 2.918 | 3.112 | 2.871 | 10.6 |
| *Eid3* | 2.491 | 2.538 | 3.735 | 6.764 |
| *Insig1* | 1.923 | 2.035 | 1.928 | 4.273 |
| *Ypel3* | 1.536 | 1.737 | 1.799 | 5.962 |

**Table S4** RMSD value of protein model and MD-optimized protein model

| Gene | Model | 0.01 v | 0.05 v | 0.5 v |
| --- | --- | --- | --- | --- |
| *Fcgr1* | 4.055 | 2.647 | 3.19 | 46.318 |
| *Hcar2* | 6.787 | 6.363 | 4.828 | 16.285 |
| *Mmp9* | 9.355 | 9.84 | 19.28 | 85.307 |
| *Lrp8* | 7.646 | 10.747 | 9.778 | 46.275 |
| *Ldlr* | 9.607 | 10.279 | 8.739 | 103.489 |
| *Eid3* | 6.664 | 9.078 | 12.087 | 38.519 |
| *Insig1* | 4.857 | 5.699 | 4.614 | 22.648 |
| *Ypel3* | 4.072 | 5.914 | 3.516 | 91.261 |
